# Metagenomic insights into microbial adaptation to the salinity gradient of a typical short residence-time estuary

**DOI:** 10.1186/s40168-024-01817-w

**Published:** 2024-06-25

**Authors:** Ziheng Wu, Minchun Li, Liping Qu, Chuanlun Zhang, Wei Xie

**Affiliations:** 1 School of Marine Sciences, Sun Yat-Sen University and Southern Marine Science and Engineering Guangdong Laboratory (Zhuhai), Zhuhai, 519082 China; 2 Department of Ocean Science and Engineering, Shenzhen Key Laboratory of Marine Archaea Geo-Omics, Southern University of Science and Technology, Shenzhen, 518055 China

**Keywords:** Salinity adaptability, Salt-in strategy, Metagenomics, Pearl river estuary

## Abstract

**Background:**

Microbial adaptation to salinity has been a classic inquiry in the field of microbiology. It has been demonstrated that microorganisms can endure salinity stress via either the “salt-in” strategy, involving inorganic ion uptake, or the “salt-out” strategy, relying on compatible solutes. While these insights are mostly based on laboratory-cultured isolates, exploring the adaptive mechanisms of microorganisms within natural salinity gradient is crucial for gaining a deeper understanding of microbial adaptation in the estuarine ecosystem.

**Results:**

Here, we conducted metagenomic analyses on filtered surface water samples collected from a typical subtropical short residence-time estuary and categorized them by salinity into low-, intermediate-, and high-salinity metagenomes. Our findings highlighted salinity-driven variations in microbial community composition and function, as revealed through taxonomic and Clusters of Orthologous Group (COG) functional annotations. Through metagenomic binning, 127 bacterial and archaeal metagenome-assembled genomes (MAGs) were reconstructed. These MAGs were categorized as stenohaline—specific to low-, intermediate-, or high-salinity—based on the average relative abundance in one salinity category significantly exceeding those in the other two categories by an order of magnitude. Those that did not meet this criterion were classified as euryhaline, indicating a broader range of salinity tolerance. Applying the Boruta algorithm, a machine learning-based feature selection method, we discerned important genomic features from the stenohaline bacterial MAGs. Of the total 12,162 COGs obtained, 40 were identified as important features, with the “inorganic ion transport and metabolism” COG category emerging as the most prominent. Furthermore, eight COGs were implicated in microbial osmoregulation, of which four were related to the “salt-in” strategy, three to the “salt-out” strategy, and one to the regulation of water channel activity. COG0168, annotated as the Trk-type K^+^ transporter related to the “salt-in” strategy, was ranked as the most important feature. The relative abundance of COG0168 was observed to increase with rising salinity across metagenomes, the stenohaline strains, and the dominant *Actinobacteriota* and *Proteobacteria* phyla.

**Conclusions:**

We demonstrated that salinity exerts influences on both the taxonomic and functional profiles of the microbial communities inhabiting the estuarine ecosystem. Our findings shed light on diverse salinity adaptation strategies employed by the estuarine microbial communities, highlighting the crucial role of the “salt-in” strategy mediated by Trk-type K^+^ transporters for microorganisms thriving under osmotic stress in the short residence-time estuary.

Video Abstract

**Supplementary Information:**

The online version contains supplementary material available at 10.1186/s40168-024-01817-w.

## Background

Microorganisms inhabiting fluctuating environments are subjected to diverse environmental stresses that can be broadly classified as physical, chemical, and biological factors. Among the various environmental stresses, salinity is characterized as a particularly influential abiotic factor in shaping microbial communities [1]. Stenohaline and euryhaline are classical categorizations used to describe the tolerance of organisms to salinity, thereby providing vital insights into their ecological niche widths in environments with a wide variation in salinity. Organisms thriving within a narrow range of salinity are regarded as stenohaline, as their life processes are confined to environments where salinity remains relatively stable. Instead, organisms capable of adapting to wide salinity fluctuations are classified as euryhaline [2, 3]. Regardless of their adaptability, microorganisms must inhabit environments that fall within specific salinity ranges to proliferate, as deviating from these ranges can disrupt metabolic processes or impede their survival. Microorganisms can be classified as stenohaline or euryhaline based on fluctuations in their relative abundances across varying salinity environments [4, 5]. Furthermore, microorganisms can also be categorized as colonizers inhabiting high-salinity or low-salinity environments based on their relative abundance patterns in response to salinity [6].

Throughout their evolutionary trajectory, prokaryotic microorganisms have developed remarkable adaptability to osmotic conditions, allowing them to thrive in diverse salinity habitats. These microorganisms have developed two distinct strategies, namely, the “salt-in” and “salt-out” mechanisms, to achieve this feat [7, 8]. Employing the “salt-in” strategy, microorganisms maintain intracellular osmolarity equilibrium with their surroundings by assimilating K^+^ while extruding Na^+^. Alternatively, those employing the “salt-out” strategy synthesize or uptake small organic compounds known as compatible solutes from the environment to prevent hazards caused by osmotic imbalance [9, 10]. Given that any disruption in osmotic pressure can lead to detrimental water influx or efflux from microbial cells, the regulation of intracellular water exchange may also be crucial for microorganisms to counteract the adverse effects of salinity stress.

As critical zones where freshwater and marine environments converge, estuaries present strong salinity gradients, making them ideal natural laboratories for studying the adaptability of microorganisms to varying salinity levels. Within an estuary, the residence time delineates the duration a water mass spends traversing a specific zone, thereby determining the temporal window for estuarine microbial community development [11]. Estuaries characterized by shorter residence times are more susceptible to be colonized by microorganisms originating from both freshwater and brackish water sources, eventually fostering the establishment of distinct microbial communities adapted to different salinity conditions [12]. This implies that distinct microbial strains, acclimatized to either low- or high-salinity environments, may be present in a short residence-time estuary.

The Pearl River Estuary (PRE) is a typical short residence-time estuary linking to the Pearl River, one of the largest rivers in southern China, to the northern South China Sea. A substantial inflow of freshwater, approximately 330 × 10^9^ m^3^ per annum, is received by the PRE, resulting in a strong salinity gradient due to the intermingling of freshwater and seawater. Taking seasonal variation into account, the estimated estuarine residence time of the PRE is relatively short, ranging from 3 to 12 days [13]. Previous studies have shown that the dominant bacterial groups within the PRE included *Proteobacteria*, *Actinobacteriota*, *Chloroflexota*, *Planctomycetota*, *Acidobacteriota*, *Nitrospirota*, *Bacteroidota*, *Spirochaetota*, *Firmicutes*, and *Gemmatimonadota*. The PRE was also reported to harbor archaeal groups such as *Euryarchaeota*, *Thaumarchaeota*, and *Crenarchaeota* [14]. Among these microorganisms, *Actinobacteriota* and *Proteobacteria* have emerged as the prevailing microbial phyla [15, 16]. *Actinobacteriota* have been found to exhibit a greater relative abundance in freshwater habitats within the coastal zone, while *Proteobacteria* dominates across the entire estuarine region [17, 18]. Given its continuous salinity gradient and short residence time, the PRE serves as an ideal natural laboratory for exploring microbial adaptation to salinity stress, both in terms of the characteristics of the estuarine microbial communities and specific microbial taxa.

Metagenomics is widely recognized as a powerful cultivation-independent paradigm for studying microbial communities. It offers a more comprehensive view compared to amplicon sequencing, primarily owing to its ability to analyze the functional capabilities of the microbial community studied [19, 20]. This broader scope is essential for understanding microbial responses and adaptations to environmental stresses. For instance, a metagenomic study of the Dead Sea revealed that halophiles in the region have adapted to high concentrations of Mg^2+^ by enriching their genomes with COGs related to Mg^2+^ transport [21]. Additionally, a metagenomic study conducted in a saltern suggested that microorganisms known to utilize the “salt-in” strategy also possess the capability to synthesize compatible solutes [22]. However, analyzing massive amount of metagenomic data poses a significant challenge for statistical analysis. To address this challenge, machine learning approaches, including the Boruta algorithm [23], have been developed and demonstrated to be particularly advantageous for biological applications [24, 25]. The Boruta feature selection algorithm employs a multiple random forest-based feature selection method that iteratively compares the importance of each feature against random predictors. Through this process, this algorithm verifies whether the classification features are statistically superior to random variables. This method is particularly advantageous when applied to metagenomic data, as it balances the sensitivity of identifying relevant variables with the control of false positive errors while saving computational resources [26].

Currently, studies in coastal ecosystems have described the taxonomic shifts of microbial communities along salinity gradients, elucidating their functional changes and community assembly mechanisms [27–31]. Fortunato et al. conducted a study in a short residence-time estuary connecting the Columbia River using metagenomics and metatranscriptomics. They found that with increasing salinity, the abundances of Na^+^ transporter genes, as well as glycine betaine and proline transporter genes, showed increasing trends. However, the total abundance of K^+^ transporters exhibited no significant trend with changes in salinity. The expression levels of Na^+^ and K^+^ transporter genes varied, not displaying any specific trend with salinity [17]. While these studies provide valuable insights into the community characteristics, functional gene abundance profiles, and gene expression patterns of microbial communities along estuarine salinity gradients, they fall short of explicitly identifying the specific genes related to microbial salinity adaptation strategies in estuarine ecosystems. Furthermore, the salinity adaptation traits of major microbial taxa inhabiting estuarine environments have not yet been fully understood. In this study, shotgun metagenomics was applied to water samples from different salinity conditions in the PRE. Based on this, taxonomic and function analyses were conducted at the metagenomic assembly and MAG levels, respectively. We aim to reveal the characteristics and mechanisms of the estuarine microbial communities in response to salinity stress and to investigate the salinity adaptation strategies of the two dominant microbial phyla *Actinobacteriota* and *Proteobacteria* in the PRE. This research will help improve our understanding of the life mechanisms of estuarine microorganisms.

## Methods

### Sample collection, DNA extraction, and metagenomic sequencing

In this study, ten surface water samples were collected from the PRE and the northern South China Sea (latitude, 21.624° N to 23.219° N; longitude, 112.809° E to 114.417° E; Additional file 1: Supplementary Figure S1) and then were filtered through 0.7 μm filter membranes during different cruises from June 2011 to October 2012. The measurement of environmental parameters (i.e., salinity, pH), DNA extraction, and shotgun metagenomic sequencing were performed as previously described [32]. Water samples were collected from shallow water layers not exceeding 20 m in depth, and salinity ranged from 0.12‰ to 34‰. All sample IDs were prefixed with “PR” (representing the Pearl River), and the numbering ascended in correlation with the rising salinity levels of their originating environmental water samples. The library samples were sequenced using the Illumina HiSeq 2000 and Illumina HiSeq 2500 platforms, which respectively generated 2 × 100 paired-end reads or 2 × 150 paired-end reads. Specifically, sample PR9 was sequenced on the Illumina HiSeq 2500 platform, while the other samples were sequenced on the Illumina HiSeq 2000 platform. After high-throughput sequencing, 11 sequenced metagenomic samples were obtained, of which PR9 and PR10 were from the same water sample, but with different sequencing depths. The sequencing depth of PR9 was deeper with 33.96 GB of sequenced data, while the sequenced data size of PR10 was 4.89 GB. The sequences from PR10 have been published, and are available under the GenBank Sequence number SRX3516207 [32]. Station locations, measured environmental parameters, and sequencing depth information are available in the supplemental information (Additional file 2: Supplementary Table S1).

### Bioinformatic processing of shotgun metagenomic data

#### Metagenomic quality control, assembly, and binning

A flexible pipeline, metaWRAP version 1.2.1, was employed to process raw metagenomic reads into metagenomic assemblies and metagenome-assembled genomes (MAGs) [33]. The raw reads were trimmed and quality-controlled with the metaWRAP-Read_qc module. Clean reads were then co-assembled and single-sample assembled using MEGAHIT version 1.1.3 [34] with kmer lengths of 21, 29, 39, 59, 79, 99, 119, and 141. All of the assembled sequences obtained were greater than 300 base pairs (bp) and were utilized for further analysis. However, only scaffolds greater than or equal to 1000 bp from the co-assembly were retained for binning (Additional file 2: Supplementary Table S2). Three metagenomic binning softwares (CONCOCT version 1.0 [35], MaxBin2 version 2.2.6 [36], and MetaBAT2 version 2.12.1 [37]) were used to produce three initial bin sets of MAGs based on co-assembled sequences in parallel. These three initial bin sets were consolidated into an improved bin set using the Bin_refinement module, setting a minimum of 50% completion and a maximum of 10% contamination, during which DAS_Tool [38], Binning_refiner [39], and metaWRAP_ Bin_refinment [33] were used. Finally, to improve the N50 contig length, increase the completeness, and reduce the contamination of the MAGs in the consolidated bin set, SPAdes version 3.13.0 [40] was used to reassemble these MAGs. Following the binning pipeline, we obtained 127 MAGs. The completion and contamination of all 127 MAGs were evaluated with CheckM version 1.1.3 (Additional file 2: Supplementary Table S3) [41]. The relative abundance of each MAG was estimated utilizing the metaWRAP-Quant_bins module with Salmon version 0.13.1 [42] based on the protocol of the metaWRAP pipeline. The relative abundance of each MAG here was expressed as genome copies per million reads. This measurement method was chosen for its comparability between metagenomes with different sequencing depth outputs and different MAG sizes (Additional file 2: Supplementary Table S4) [33]. The percentage of sequences recruited by these MAGs from the co-assembly, as well as the proportion mapped to each metagenomic sample, were calculated using CoverM version 0.6.1 (“genome” mode with default settings, https://github.com/wwood/CoverM) with minimap2 version 2.26-r1175 [43] and SAMtools version 1.19 [44] (Additional file 2: Supplementary Table S5).

#### Taxonomic assignment of the metagenomes

Two methods were employed for taxonomic annotation of the metagenomes. Contigs of each metagenome were taxonomically classified against the NCBI RefSeq database (2023–5-10, downloaded from kaiju.binf.ku.dk), using Kaiju version 1.9.2 with default settings [45]. Kaiju performed a database search based on amino acid sequence similarity and provided classification results at the species level. Sequences annotated as viruses or unclassified were excluded from the output results, and the relative abundances of the remaining classified sequences were recalculated (Additional file 2: Supplementary Table S6). Barrnap version 0.9 (https://github.com/tseemann/barrnap/) was used for rRNA gene sequence retrieval based on single-sample assembled contigs, and the 16S rRNA gene fragments (i.e., 16S miTags) were then extracted. The 16S rRNA gene sequences were classified by the RDP (Ribosomal Database Project) classifier. The classification was performed under an 80% confidence threshold using the 16S rRNA training set 18 (Additional file 2: Supplementary Table S7) [46].

#### Functional annotation of the metagenomes and the MAGs

Functional annotation was performed at the open reading frame level for the assembled contigs of 11 metagenomes and 127 MAGs. Prodigal version 2.6.3 [47] was used for gene prediction, and eggNOG version 5.0 database [48] was used for functional annotation using the DIAMOND version 0.9.14 [49] with eggNOG-mapper-2.1.6 [50] (E-value = 0.001). The metagenome mode setting was used for the metagenomes (Additional file 2: Supplementary Table S2), and the genome option (-itype genome) was used for the MAGs.

#### GTDB classification of the MAGs and phylogenetic analysis

GTDB-Tk version 1.3.0 was used to classify the MAGs generated from this study as well as to build phylogenetic trees based on the Genome Taxonomy Database (GTDB, release 95) [51, 52]. Taxonomy designation of the MAGs was performed using the classify_wf workflow within the GTDB-Tk. The classification and phylogenetic analysis of archaeal MAGs were performed based on 122 archaeal marker genes, and 120 bacterial marker genes were used to classify bacterial MAGs [53]. FastTree version 2.1.10 [54] was used to infer the phylogenetic trees of MAGs with the maximum-likelihood model based on the multiple sequence alignments provided by the GTDB-Tk align module. The bootstrap value of each branch was calculated with the Shimodaira-Hasegawa test based on 1000 replicates. The taxonomic assignment results for MAGs can be found in Additional file 2: Supplementary Table S3, S4, and S5. The taxonomic annotation of COG0168 was conducted by integrating the annotation results from the eggNOG v5.0 database and aligning against the NCBI_nr database using BLASTp (E-value < 2e-28) [55]. The multiple sequence alignment based on amino acid sequences of COG0168 was created with Clustal-Omega version 1.2.4 [56]. FastTree version 2.1.10 was again used to infer the phylogenetic tree with the default setting based on the provided multiple sequence alignment. All the phylogenetic trees in this paper were drawn and presented using iTOL version 6.6 [57].

### Statistical analysis

Statistical analyses and data visualization were carried out using the R language version 4.1.1 (https://www.R-project.org/) [58], ImageGP online platform (http://www.ehbio.com/ImageGP/) [59], and Hiplot Pro online platform (https://hiplot.com.cn/).

All of the statistical tests were performed using R language version 4.1.1. For multiple group comparisons, we performed the Kruskal–Wallis rank-sum test (K-W test). If the Kruskal–Wallis rank-sum test was significant (*p* ≤ 0.05), we then performed Dunn’s multiple comparison tests with Bonferroni adjustment. Dunn’s multiple comparison tests were done using the R package dunn.test version 1.3.5. The Shannon–Wiener diversity index calculation, hierarchical clustering analysis, Bray–Curtis dissimilarity-based non-metric multidimensional scaling (NMDS), and analysis of similarities (ANOSIM) were all conducted using the R package vegan version 2.5–7 (https://CRAN.R-project.org/package=vegan). In this study, the Bray–Curtis dissimilarity was computed based on the relative abundances of MAGs or COGs. The Shannon–Wiener diversity index of each sample was calculated at the species level based on the taxonomic annotation results from Kaiju software. In R, the lm() function and package ggplot2 version 3.4.2 [60] were used for linear regression, and regression parameters were obtained using the summary() function. To further enhance the statistical robustness of our results, the R package boot version 1.3–28 was employed to conduct bootstrap tests on the *p* values of the Kruskal–Wallis rank-sum tests, Dunn’s significance tests, and the statistics of the linear regressions (1000 resamplings) [61]. The significance criteria (alpha) for simple linear regressions were all set to 0.05. The sampling map was drawn by Ocean Data View version 5.6.3 (https://odv.awi.de/).

### Metagenomes and MAGs categorization

Metagenomes and MAGs were both categorized by salinity. Metagenomes PR1, PR2, PR3, and PR4 were from stations with salinities below 10‰ and were classified as low-salinity metagenomes. Metagenomes PR5, PR6, and PR7 were from stations with salinities ranging from 10‰ to 30‰ and were classified as intermediate-salinity metagenomes. Metagenomes PR8, PR9, PR10, and PR11 were from stations with salinities above 30‰ and were classified as high-salinity metagenomes (Additional file 2: Supplementary Table S1).

To elucidate the genomic features of the obtained MAGs across the salinity gradient, we employed the following classification scheme. If the average relative abundance of a specific MAG in a particular salinity category (indicated as C-salinity, where C represents one of the following: low, intermediate, or high) surpassed the average abundance in the other two categories by an order of magnitude, then this MAG was classified as stenohaline, indicating its adaptability to the C-salinity level. MAGs failing to meet this classification criterion were categorized as euryhaline MAGs. Further subdivision of these euryhaline MAGs was based on their average relative abundances across metagenomes of different salinity categories, resulting in the identification of three subsets: high-, intermediate-, and low-salinity inclined euryhaline MAGs (Additional file 2: Supplementary Table S4).

### Feature selection

To identify the characteristic COGs of MAGs associated with the different salinity categories, we applied the Boruta feature selection algorithm based on random forest variable importance measures, implemented in version 7.0.0 of the Boruta R package. Specifically, the Boruta function was executed utilizing a maximum of 8000 runs and confidence level of 0.05 [25]. After up to 8000 importance source runs, no COGs were left tentative. Boruta first created a corresponding “shadow” COGs matrix, whose relative abundances were obtained by randomly scrambling the original relative abundances of COGs between objects and then ran a random forest classifier to gather the importance score of each COG [23]. By iteratively fitting the random forest model, Boruta tested whether each COG was significantly more important than the “shadow” COG until all the COGs were classified as “confirmed” or “rejected.”

## Results

### Community structure and diversity of prokaryotes across the salinity gradient

**Fig. 1:**
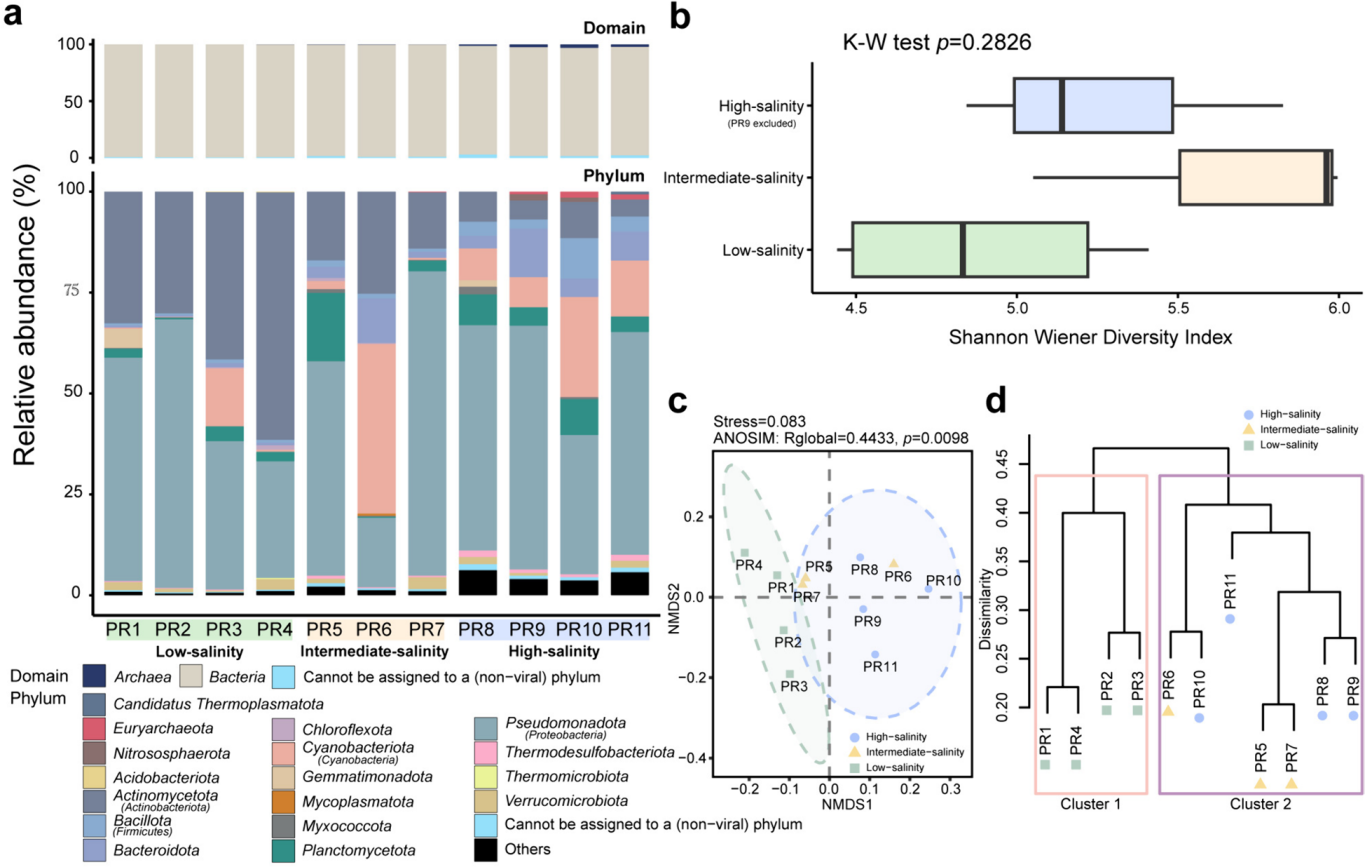
Taxonomic profiles and functional patterns of the microbial communities across the salinity gradient. **a** Relative abundances of microbial domains (above) and the top 10 most abundant phyla (below) in each metagenome obtained from the taxonomic assignments of contigs. Boxplot components, center lines, medians; box limits, 25^th^ and 75^th^ percentiles; whiskers, 1.5 × interquartile range from the 25^th^ and 75^th^ percentiles. Source data are provided in Additional file 2: Supplementary Table S6. **b** Comparison of Shannon–Wiener diversity index distributions estimated for the three salinity categories. The number of metagenomes included in each category is as follows: low-salinity category, *n* = 4; intermediate-salinity category, *n* = 3; high-salinity category, *n* = 3 (PR9 was excluded). Due to the limited number of samples across different salinity categories, bootstrap testing was not conducted. Source data are provided in Additional file 2: Supplementary Table S6. **c** NMDS performed on the Bray–Curtis dissimilarities of COG relative abundance profiles of the 11 metagenomic samples from this study. The ellipses in the plot mark the 90% confidence interval for metagenomes grouped by the salinity category. **d** Complete linkage hierarchical clustering based on the relative abundances of COGs in the metagenomes using Bray–Curtis dissimilarities. The salinity categories of the metagenomes are denoted using markers of different colors and shapes. Different colored boxes indicate the grouping of these branches into two main subdivisions

After quality control filtering, the 11 metagenomes from this study, a total of 271,182,520 reads were retained for further analysis, yielding an average of 24,652,956 reads per metagenome. The reads were subsequently assembled into contigs, with the resulting number of contigs ranging from 15,124 to 349,586 for the different metagenomes. The average N50 contig length was 2718 bp (Additional file 2: Supplementary Table S2). For taxonomic profiling, the entire set of contigs from 11 metagenomes, totaling 616,372 contigs (i.e., all assembled contigs having a length greater than 300 bp), were annotated with Kaiju. Among these, 260,006 sequences were annotated at the species level for prokaryotes, accounting for 42.18% of all contigs. The remaining sequences included 15,703 viral sequences, 278,087 unclassified sequences, and 62,576 sequences that could not be assigned to a (non-viral) species. Based on the contig-level Kaiju annotation, 55 prokaryotic phyla were annotated, with 46 attributed to *Bacteria* and 9 to *Archaea* (Additional file 2: Supplementary Table S6). Barrnap extracted 453 16S miTags from the metagenomes, with 314 of them being classified as either *Archaea* or *Bacteria* at a confidence threshold of 80%. These 16S miTags were classified into 14 phyla, of which 11 were affiliated with *Bacteria* and 3 with *Archaea* (Additional file 1: Supplementary Figure S2 and Additional file 2: Supplementary Table S7). In each sample, bacterial taxa were predominant. Results from both methods showed that *Proteobacteria* was the most abundant bacterial phylum, followed by *Actinobacteriota* and *Cyanobacteria*. Additionally, *Bacteroidota* and *Planctomycetota* had high relative abundances in certain metagenomes. *Proteobacteria* dominated across almost all salinity categories, while the relative abundance of *Actinobacteriota* decreased with increasing salinity. Archaeal taxa were primarily annotated in metagenomes of high-salinity categories, exhibiting relatively low relative abundances in metagenomes of low- and intermediate-salinity categories. *Euryarchaeota* emerged as the predominant archaeal phylum, and it was annotated in every sample (Fig. 1a and Additional file 1: Supplementary Figure S2a). Based on the annotations provided by Kaiju on the taxonomic profiles of the contigs, the results from NMDS and ANOSIM analyses indicated a significant difference in taxonomic composition among different salinity categories (Additional file 1: Supplementary Figure S2b; NMDS, stress = 0.053; ANOSIM, *p* = 0.001; number of permutations = 99,999). The results of the hierarchical clustering were displayed through a dendrogram, which was divided into four branches: low-salinity samples PR2 and PR3 were grouped together; low-salinity samples PR1 and PR4 formed another group; all high-salinity samples constituted a separate group; and all intermediate-salinity samples formed yet another group. The mean Bray–Curtis distance was 0.623 (Additional file 1: Supplementary Figure S2c).

The alpha diversity of each microbial community was assessed using the Shannon–Wiener diversity index at the species level, with taxonomic annotations derived from Kaiju software. The alpha diversity indices of these samples ranged from 4.44 and 6.00, with an average of 5.28. Specifically, the average values for low-, intermediate-, and high-salinity categories were 4.88, 5.70, and 5.39, respectively. However, no significant difference was detected among the three salinity categories, as determined by the Kruskal–Wallis rank-sum test (Fig. 1b, *p* = 0.2826). To fulfill the independence assumption of the Kruskal–Wallis rank-sum test, PR9 was excluded from this test.

### Functional characteristics of the metagenomes across the salinity gradient

Functional dissimilarities between samples were quantified using Bray–Curtis dissimilarity, resulting in a mean Bray–Curtis distance of 0.352 (Fig. 1c and d). NMDS and ANOSIM analyses revealed a significant difference in the functional gene composition across metagenomes of different salinity categories (NMDS, stress = 0.083; ANOSIM, *p* = 0.0098; number of permutations = 99,999). To depict the range of variation for the high-salinity and low-salinity metagenomes, 90% confidence ellipse intervals were plotted. The intermediate-salinity metagenomes fell within the variation range of the high-salinity metagenomes but were excluded from that of the low-salinity metagenomes (Fig. 1c). Using complete linkage hierarchical clustering, a dendrogram comprising 11 branches was constructed, and these branches were grouped into two main subdivisions (Fig. 1d). Unsupervised hierarchical clustering revealed that the high-salinity and intermediate-salinity metagenomes were distinct from the low-salinity metagenomes. These findings suggested that the dissimilarities between low- and high-salinity metagenomes were more pronounced than those between intermediate- and high-salinity metagenomes.

### Genome reconstruction with metagenomic binning

By employing metagenomic binning, we successfully assembled 127 MAGs of medium quality (completeness ≥ 50%, contamination ≤ 10%, totaling 102) and high quality (completeness ≥ 90%, contamination ≤ 5%, totaling 25), as previously established quality definitions (Additional file 2: Supplementary Table S3) [62]. The read coverages of these MAGs varied from 3.92% to 21.67% per metagenomic sample, averaging 10.63%. In total, these MAGs comprised 0.27 Gbp and recruited 21.79% of the sequences from the co-assembly. Specifically, out of the 650,161 total contigs from the co-assembly, 141,665 sequences were successfully mapped (Additional file 2: Supplementary Table S5). The completeness of these MAGs ranged from 50.13% to 98.39%, with contamination ranging from 0% to 9.89%. Taxonomic classification revealed that the prevailing majority (116 MAGs) were ascribed to the domain *Bacteria*, while a mere 11 MAGs were attributed to the domain *Archaea*. These MAGs spanned 12 bacterial and 3 archaeal phyla, and their detailed characteristics are provided in Additional file 2: Supplementary Table S3.

**Fig. 2:**
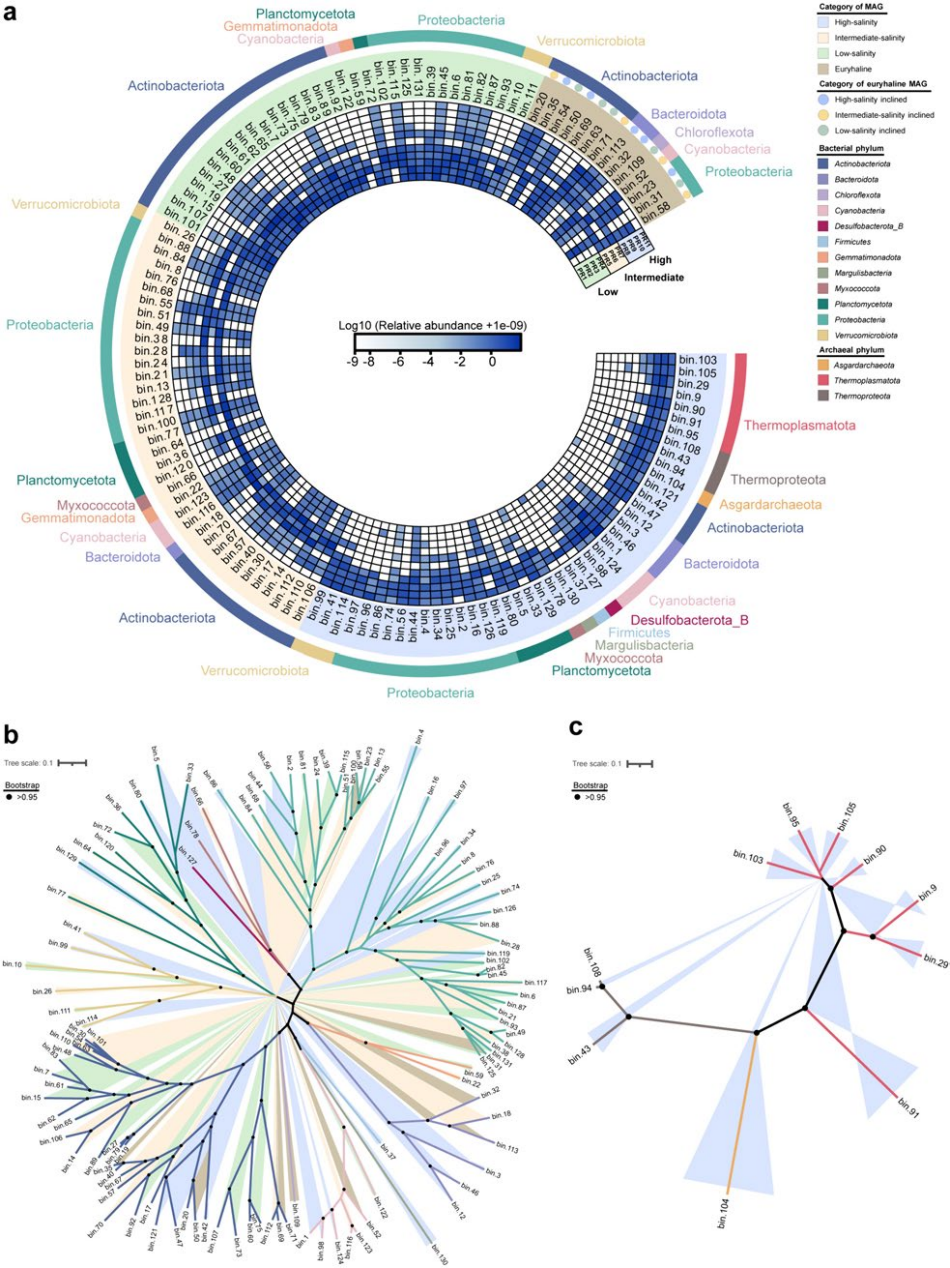
Relative abundances and phylogenetic relationships of MAGs. **a** Heatmap representing the log10-transformed relative abundances of MAGs. Abundance values were computed as genome copies per million reads. To avoid the log transformation of zero values, a small constant (i.e., 1e-9) was added to the raw relative abundance of each MAG. Detailed information of the MAG abundance per metagenomic sample can be found in Additional file 2: Supplementary Table S4. Background color indicates the salinity category, while the outer color bar denotes the phylum. **b, c** Phylogenetic trees of the reconstructed bacterial and archaeal MAGs. The different background and branch colors in the trees represent the salinity categories and phylum taxonomic annotations of the MAGs, respectively. Color codes are the same as in part (**a**) of the figure. The solid black dots label branches with a bootstrap value greater than 0.95

The output of metagenomic binning was consistent with the previously obtained results on prokaryotic community composition, thereby demonstrating the robustness of our findings. Among the bacterial MAGs, the phyla *Proteobacteria* (43 MAGs) and *Actinobacteriota* (37 MAGs) were dominant, followed by *Planctomycetota* (9 MAGs), *Cyanobacteria* (7 MAGs), *Bacteroidota* (6 MAGs), and *Verrucomicrobiota* (6 MAGs), while the remaining MAGs belonged to miscellaneous bacterial phyla. Archaeal MAGs were assigned to three phyla, namely *Thermoplasmatota* (7 MAGs), *Thermoproteota* (3 MAGs), and *Asgardarchaeota* (1 MAG). The relative abundances of these MAGs varied across the metagenomes of different salinity categories. While the bacterial MAGs were widely distributed among all metagenomes, the archaeal MAGs were mainly found in the intermediate- and high-salinity metagenomes, being particularly more abundant in the latter. Applying the classification rule outlined in the Methods section, a total of 33 MAGs were classified as low-salinity stenohaline, 36 MAGs as intermediate-salinity stenohaline, and 44 MAGs as high-salinity stenohaline (including 11 archaeal MAGs). Moreover, 14 MAGs were classified as euryhaline. Notably, the two dominant phyla *Actinobacteriota* and *Proteobacteria* jointly constituted 71 stenohaline MAGs, accounting for 63% of the total stenohaline MAGs. Additionally, these two phyla encompassed nine euryhaline MAGs, representing 64% of the total euryhaline MAGs. Within the *Ilumatobacteraceae* family of the *Actinobacteriota* phylum, five out of seven MAGs were categorized as intermediate-salinity stenohaline. The other two belonged to the low-salinity stenohaline and high-salinity inclined euryhaline categories. In the family *Mycobacteriaceae*, excluding two MAGs of the genus *Aquiluna* classified as intermediate-salinity stenohaline, the remaining four were categorized as low-salinity stenohaline. Furthermore, all four MAGs in the family *Nanopelagicaceae* and the order *Solirubrobacterales* were classified as low-salinity stenohaline. In another major microbial group, the phylum *Proteobacteria*, within the family *Rhodobacteraceae*, apart of two MAGs classified in the euryhaline category (one as intermediate-salinity inclined and the other as high-salinity inclined), the remaining MAGs were categorized as intermediate-salinity stenohaline. All MAGs in the family *Puniceispirillaceae* were classified as high-salinity stenohaline. MAGs in the families *Burkholderiaceae* and *Beijerinckiaceae* were primarily categorized as either low-salinity stenohaline or intermediate-salinity stenohaline, whereas MAGs in the family *Ga0077536* were mainly classified as either intermediate-salinity stenohaline or high-salinity stenohaline (Fig. 2, Additional file 2: Supplementary Table S4).

NMDS and ANOSIM were performed to evaluate the statistical difference in the composition of MAGs between different stenohaline categories, ensuring stable and reliable classification results. As depicted in Additional file 1: Supplementary Figure S3, both NMDS and ANOSIM confirmed that the stenohaline categories were significantly different from one another. This significant difference was observed not only among the three stenohaline categories, but also in each pairwise comparison (NMDS, stress = 0.145; ANOSIM, *p* = 0.001, number of permutations = 99,999).

### Functional profile of the reconstructed MAGs

Based on the relative abundances of COGs annotated in the MAGs, a hierarchical clustering analysis using the Bray–Curtis dissimilarity was conducted (Additional file 1: Supplementary Figure S4). The average Bray–Curtis dissimilarity was 64% and the generated dendrogram demonstrated two distinct clusters of the archaeal and bacterial MAGs at the domain level. However, compared with the finding at the metagenome level across the salinity gradient (Fig. 1d), no clear patterns based on salinity categories were observed at the MAG level. The heterogeneity observed in the abundance composition of COGs within MAGs suggested that taxonomic distinctions play a crucial role in differentiating MAGs. Additionally, characteristic genes associated with adaptation to salinity or other estuarine environmental factors could also be influencing such clustering analysis.

### Feature selection for the stenohaline bacterial MAGs

**Fig. 3:**
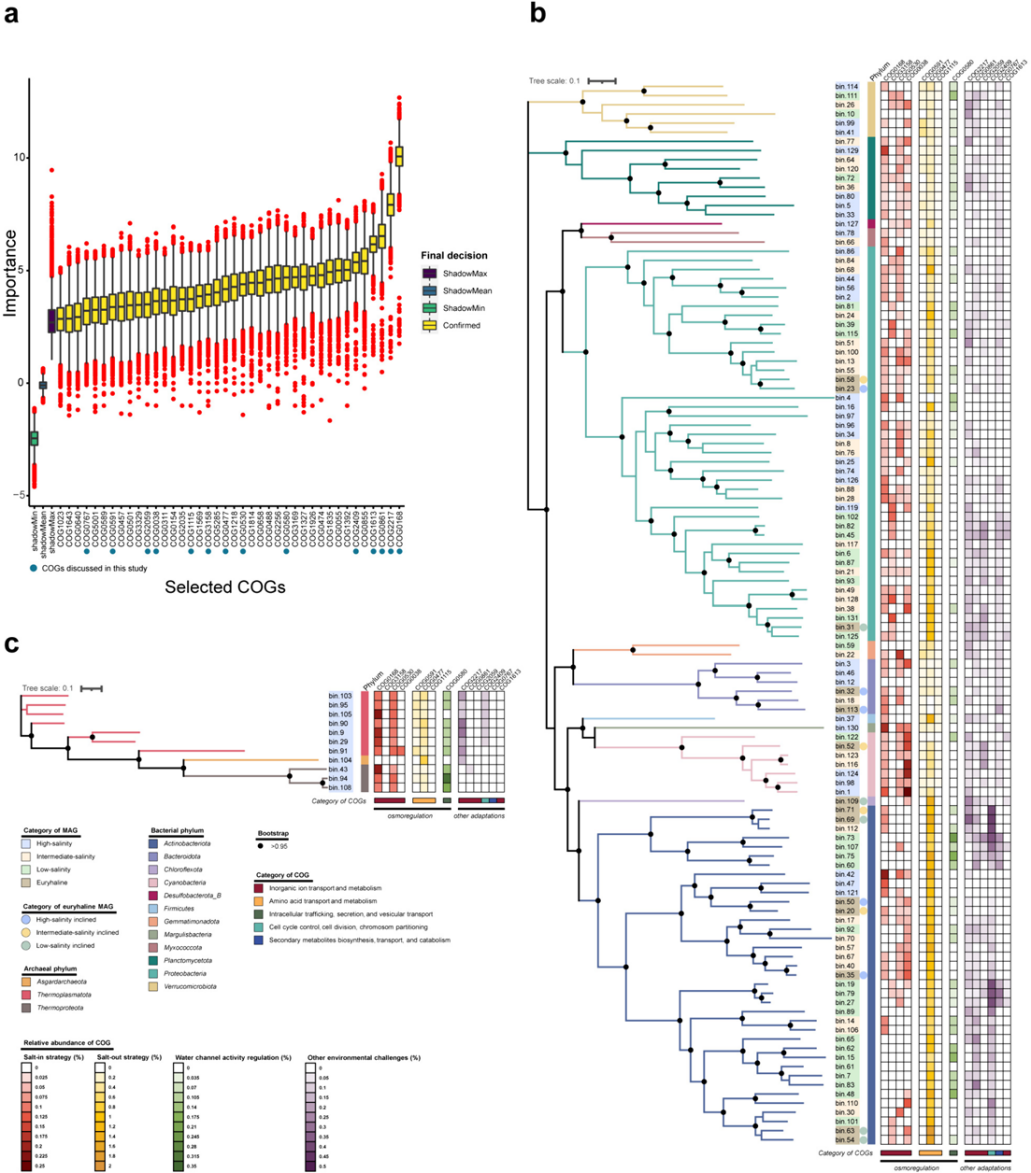
Feature importances and relative abundances of selected COGs. **a** Featured importances of the selected COGs. Each boxplot represents the importance values computed from up to 8000 permutations. The solid blue dots highlight the COGs that are discussed in the text and shown in (**b**) and (**c**). ShadowMax, maximum score of the shadow matrix; ShadowMean, mean score of the shadow matrix; ShadowMin, minimum score of the shadow matrix. Boxplot components, center lines, medians; box limits, 25^th^ and 75^th^ percentiles; whiskers, 1.5 × interquartile range from the 25^th^ and 75^th^ percentiles; red dots, outliers. **b, c** Heatmaps representing the relative abundances of the discussed COGs in bacterial and archaeal MAGs, and the phylogenetic trees. The background color of each MAG ID represents its salinity category while the color bars preceding the heatmaps represents its phylum affiliation. The euryhaline MAGs of different salinity inclinations are marked by dots of different colors. The functional categories of the COGs are indicated by the color bars below the heatmaps. The solid black dots label branches with a bootstrap value greater than 0.95. Source data are provided in Additional file 2: Supplementary Table S10

The genomic profiles of the MAGs were characterized by their COG compositions [63]. We hypothesized that microorganisms adapted to different salinity environments exhibit different patterns in the relative abundances of certain COGs related to salinity adaptation. As such, these COGs may play a crucial role in distinguishing MAGs of various salinity categories. However, owing to the divergence between *Bacteria* and *Archaea*, some COGs related to phylogeny may interfere with the identification of COGs associated with salinity adaptation. Thus, we excluded archaeal MAGs from the selection model but still retained them for further analysis. Employing the Boruta feature selection algorithm, 40 out of 12,612 COGs were identified as important in distinguishing the low-, intermediate-, and high-salinity MAGs (Fig. 3a). Although seven of these COGs were categorized as “function unknown,” the largest subset (13 COGs) within these 40 COGs fell into the “inorganic ion transport and metabolism” category. Additionally, other categories including “secondary metabolites biosynthesis, transport and catabolism,” “amino acid transport and metabolism,” and “replication, recombination and repair” each contained three COGs, while the remaining COG categories only comprised one or two COGs each (Additional file 2: Supplementary Table S8). Here, we have selected COG0168, COG0580, COG0530, COG0477, COG3158, COG1115, COG0038, COG0591, COG2217, COG0861, COG2059, COG2409, COG0767, and COG1613 for further discussion, as they were considered to potentially relate to microbial adaptation to environmental stresses in the estuarine region.

### COGs associated with microbial adaptation to salinity

Among these 40 COGs selected by the Boruta algorithm, COG0168 (a low-affinity K^+^ transport system that interacts with K^+^ uptake protein TrkA of the Trk-type K^+^ transport system), COG0530 (Ca^2+^: K^+^/Na^+^ antiporter), COG3158 (K^+^ transmembrane transporter activity), COG0038 (Cl^−^ channel), COG0591 (symporter activity), COG0477 (major facilitator superfamily), COG1115 (amino acid carrier protein), and COG0580 (water channel activity) were considered to be closely related to the microbial salinity adaptation (Fig. 3). These COGs represented various microbial strategies for salinity adaptation, highlighting the diverse osmoregulation mechanisms of the microbial communities in the PRE (Additional file 2: Supplementary Tables S8, S9, and S10) [7]. In this context, the COGs related to the “salt-in” strategy were referred to as SIR COGs, while those related to the “salt-out” strategy were referred to as SOR COGs. According to the importances assessed by the Boruta algorithm, these COGs were ranked in descending order as follows: COG0168, COG0580, COG0530, COG0477, COG3158, COG1115, COG0038, and COG0591 (Fig. 3a). In the phylum *Actinobacteriota*, COG0477 was consistently found in high abundance across all salinity categories, with a notable absence of COG0168 in low-salinity MAGs. For instance, within the family *Mycobacteriaceae*, only intermediate-salinity MAGs were annotated with COG0168. In another major microbial phylum, the *Proteobacteria*, COG0168 was widely annotated in the intermediate-salinity, high-salinity, and euryhaline categories, but only two low-salinity MAGs (bin6 and bin125) were annotated. COG0477 was also frequently annotated, except for bin4 (high-salinity category). However, COG0530 was not annotated in low-salinity MAGs. In the family *Rhodobacteraceae* (a major marine bacterial group), annotations of COG0168, as well as COG0591 and COG0477, were found in all MAGs (Fig. 3b). For the archaeal MAGs, except for bin104 (classified under the phylum *Asgardarchaeota*), annotations of COG0168 and COG0530, alongside COG0591 and COG0477, were present in the remaining MAGs. Excluding bin104 and bin105, annotations of COG0580, associated with the regulation of water channel activity, were found in all archaeal MAGs (Fig. 3c).

**Fig. 4:**
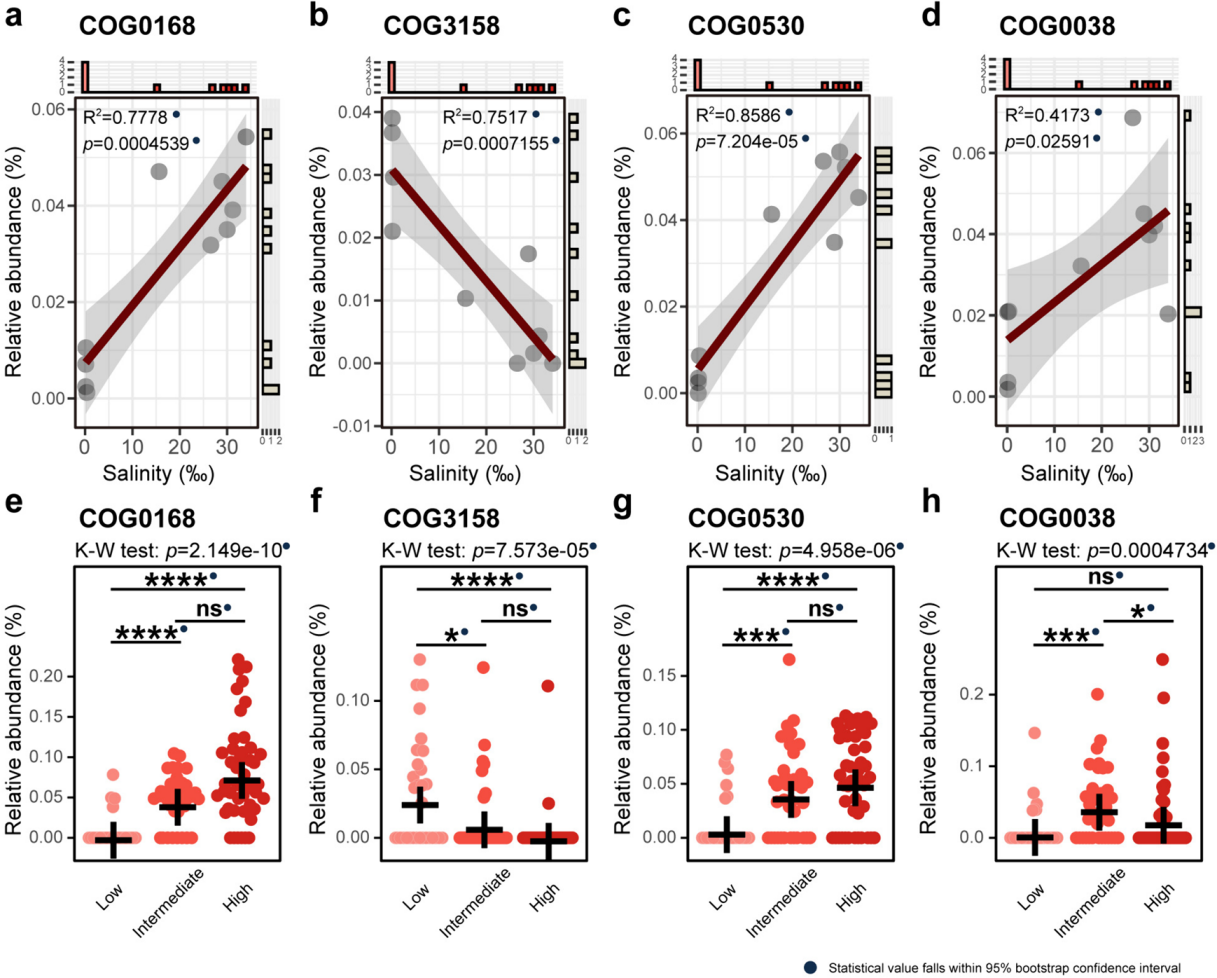
Relative abundances of the SIR COGs in metagenomes and stenohaline MAGs. **a–d** Relative abundance trends of COG0168, COG3158, COG0530, and COG0038 at the metagenome level. The regression R-squared values and *p* values are embedded within the graphs. **e–h** Relative abundances of the four SIR COGs in stenohaline MAGs. All R-squared values and *p* values fall within the 95% confidence interval of bootstrap tests and are marked with solid blue dots. Number of MAGs involved in statistical calculations: low-salinity category, *n* = 33; intermediate-salinity category, *n* = 36; high-salinity category, *n* = 44. The crosses in the figures indicate the mean relative abundances. Statistical significance symbols: ****p ≤ 0.0001, ***p ≤ 0.001, **p ≤ 0.01, *p ≤ 0.05, ns *p* > 0.05 (Kruskal–Wallis rank-sum test with Dunn’s multiple comparison test). Source data are provided in Additional file 2: Supplementary Tables S9 and S10

**Fig. 5:**
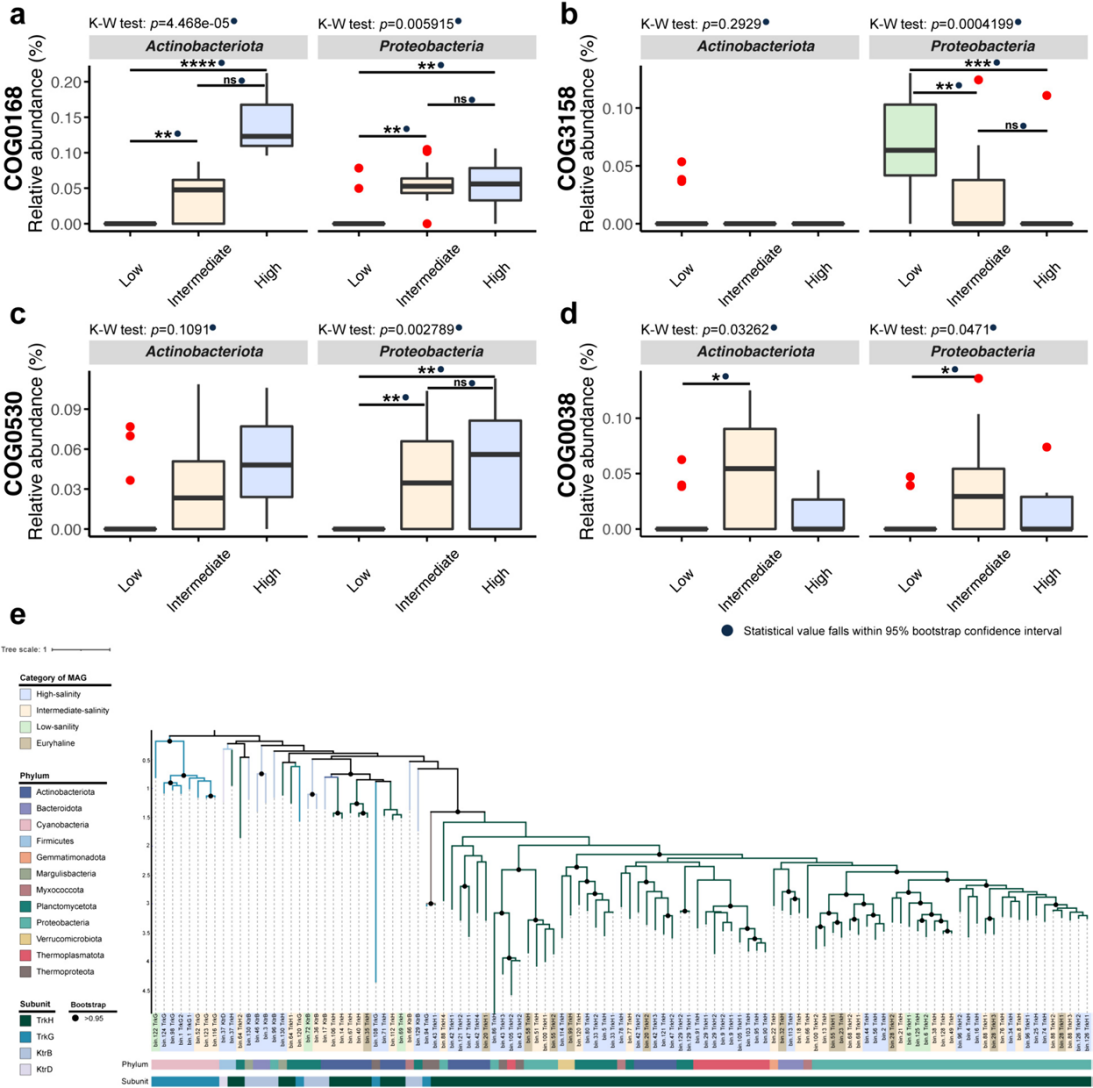
Relative abundances of the SIR COGs in *Actinobacteriota* and *Proteobacteria* stenohaline MAGs and phylogeny of COG0168. **a–d** Relative abundances of the SIR COGs in stenohaline MAGs affiliated with *Actinobacteriota* and *Proteobacteria*. All *p* values fall within the 95% confidence interval of bootstrap tests and are marked with solid blue dots. Number of MAGs involved in statistical calculations: low-salinity category *Actinobacteriota*, *n* = 17; low-salinity category *Proteobacteria*, *n* = 11; intermediate-salinity category *Actinobacteriota*, *n* = 10; intermediate-salinity category *Proteobacteria*, *n* = 16; high-salinity category *Actinobacteriota*, *n* = 3; and high-salinity category *Proteobacteria*, *n* = 13. Boxplot components, center lines, medians; box limits, 25^th^ and 75^th^ percentiles; whiskers, 1.5 × interquartile range from the 25^th^ and 75^th^ percentiles; red dots, outliers. Statistical significance symbols: ****p ≤ 0.0001, ***p ≤ 0.001, **p ≤ 0.01, *p ≤ 0.05, ns *p* > 0.05 (Kruskal–Wallis rank-sum test with Dunn’s multiple comparison test). Source data are provided in Additional file 2: Supplementary Table S10. **e** Phylogenetic tree constructed based on the amino acid sequences of COG0168 from all MAGs. Different background colors of the labels indicate different salinity categories, while the color bars indicate the microbial phyla and subunit types. The solid black dots label branches with a bootstrap value greater than 0.95

**Fig. 6:**
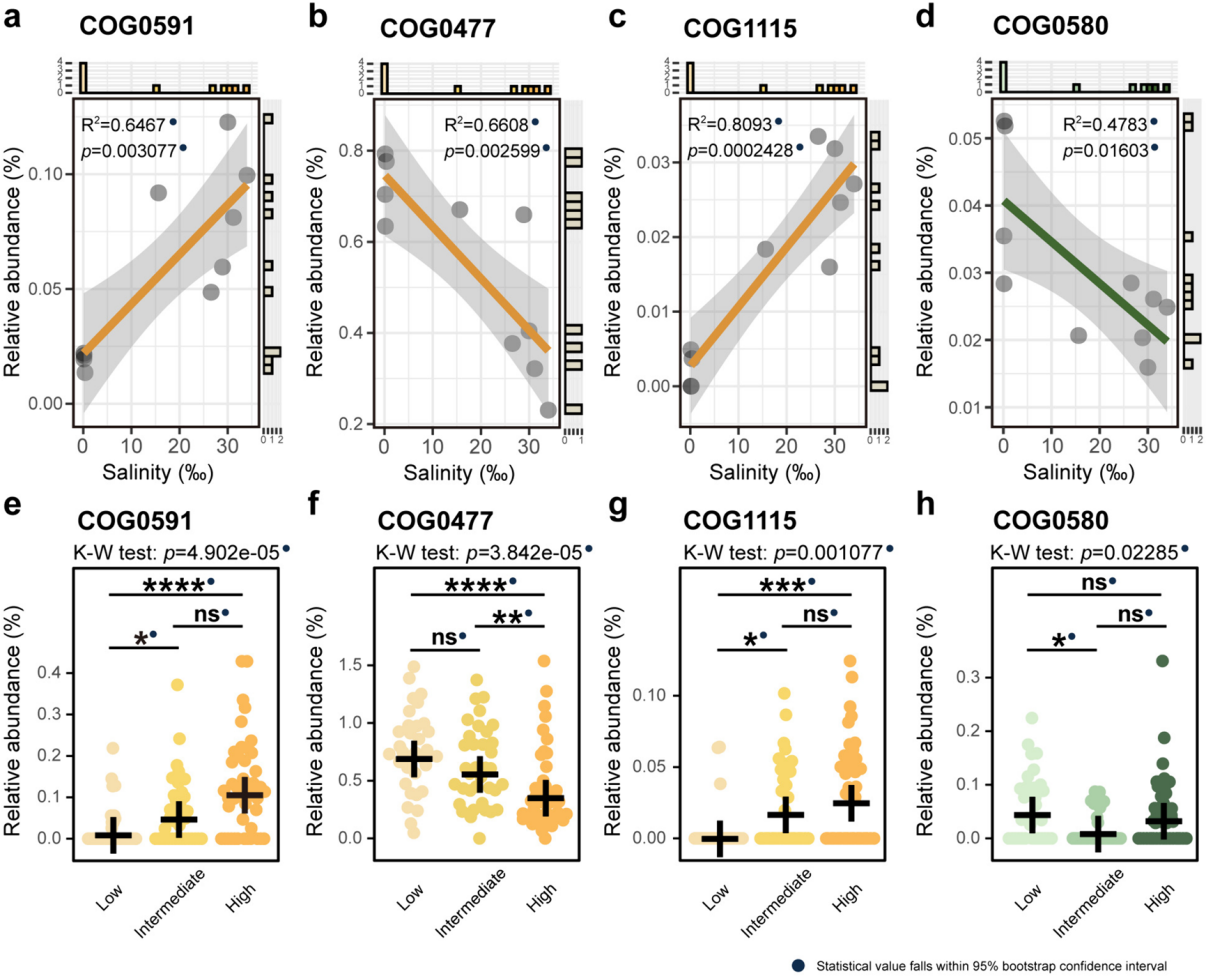
Relative abundances of the SOR COGs and COG0580. **a–d** Relative abundance trends of the SOR COGs and COG0580 at the metagenome level (R-squared values and *p* values shown in the plots). **e–h** Relative abundances of the SOR COGs and COG0580 in stenohaline MAGs. All *R*-squared values and *p* values fall within the 95% confidence intervals from bootstrap tests (solid blue dots). Number of MAGs involved in statistical calculations: low-salinity category, *n* = 33; intermediate-salinity category, *n* = 36; high-salinity category, *n* = 44. The crosses in the figures indicate the mean relative abundances. Statistical significance symbols: ****p ≤ 0.0001, ***p ≤ 0.001, **p ≤ 0.01, *p ≤ 0.05, ns *p* > 0.05 (Kruskal–Wallis rank-sum test with Dunn’s multiple comparison test). Source data are provided in Additional file 2: Supplementary Table S10

In the subsequent sections, we investigated the abundance patterns of SIR COGs and SOR COGs (Figs. 4, 5, and 6), as well as COG0580 (regulation of water channel activity) at both the metagenome and MAG levels (Fig. 6, Additional file 1: Supplementary Figure S5, Additional file 2: Supplementary Table S9, Additional file 2: Supplementary Table S10).

#### The “salt-in” strategy

When facing elevated salinity, microorganisms have demonstrated their adaptive capabilities by regulating inorganic ion transport, known as the “salt-in” strategy. This adaptive approach is characterized by the transport of vital ions, such as K^+^, Ca^2+^, and Cl^−^, which may play a critical role in maintaining cellular homeostasis under salt-induced stress [7, 64, 65]. Considering the functional roles of COG0168 (Trk-type K^+^ transport system), COG3158 (Kup-type K^+^ transport system), COG0530 (Ca^2+^: K^+^/Na^+^ antiporter), and COG0038 (Cl^−^ channel), these were assumed to be implicated in the “salt-in” strategy. In our study, the relative abundance profiles of these COGs exhibited different characteristics with changes in salinity.

##### The “salt-in” strategy of the estuarine microbial community

Our analyses revealed distinct patterns in the relative abundances of COG0168 (Fig. 4a, *R*^2^ = 0.7778, *p* = ), COG3158 (Fig. 4b, *R*^2^ = 0.7517, *p* = 0.0007155), COG0530 (Fig. 4c, *R*^2^ = 0.8586, *p* = 0.00010327.204e-05), and COG0038 (Fig. 4d, *R*^2^ = 0.4173, *p* = 0.02591), in response to increasing environmental salinity. Specifically, COG0168, COG0530, and COG0038 exhibited an upward trend, while COG3158, annotated as the Kup-type K^+^ transport system, displayed a linear decline with rising salinity. The research findings concerning stenohaline MAGs also uncovered significant differences in the relative abundances of these four COGs across different salinity categories (Kruskal–Wallis rank-sum test: *p* < 0.05, Fig. 4e–h). COG0168 and COG0530 exhibited similar abundance patterns and showed significant differences between the high- and low-salinity categories, as well as between the intermediate- and low-salinity categories. Moreover, these two COGs showed the highest average relative abundances in the high-salinity category, followed by the intermediate-salinity category, but were almost negligible in the low-salinity category (Fig. 4e and g). In contrast, COG3158 exhibited the highest average relative abundance in the low-salinity category, and significant differences were also observed between the high- and low-salinity categories, and the intermediate- and low-salinity categories (Fig. 4f). COG0038 showcased a distinctive pattern, with the highest average relative abundance being observed in the intermediate-salinity category, followed by the high-salinity category and the low-salinity category. Significant differences in relative abundance were found between the high- and intermediate-salinity categories, as well as between the intermediate- and low-salinity categories (Fig. 4h).

##### The “salt-in” strategy of the estuarine dominant microbial phyla

We further investigated the differences in the relative abundances of these COGs in MAGs belonging to the two dominant phyla, *Actinobacteriota* and *Proteobacteria*. In both phyla, the high-salinity category demonstrated a significantly higher relative abundance of COG0168 compared with the low-salinity category, with a similarly significant difference observed between the intermediate- and low-salinity categories (Fig. 5a). In the phylum *Actinobacteriota*, the annotations of COG3158 were confined to a small subset of the low-salinity MAGs. In contrast, within the phylum *Proteobacteria*, a significant difference in the relative abundance of COG3158 was observed between the intermediate- and low-salinity categories, as well as between the high- and low-salinity categories. For each phylum, the average relative abundance of COG3158 was higher in the low-salinity category (Fig. 5b). The relative abundance of COG0530 in the phylum *Actinobacteriota* showed no significant difference across salinity categories. However, in the phylum *Proteobacteria*, the relative abundance of this COG was significantly higher in both the high- and intermediate-salinity categories than in the low-salinity category (Fig. 5c). As for COG0038, significant differences were observed solely between the intermediate- and low-salinity categories in these two phyla, with a higher relative abundance in the intermediate-salinity category (Fig. 5d).

##### Phylogenetic analysis of COG0168

COG0168 represents the Trk-type K+ transport system, which is one of the main K^+^ transport systems in microorganisms [7, 66]. Using the Boruta feature selection model, this COG was identified as the top-ranked in terms of importance. Phylogenetic analysis was conducted on the amino acid sequences of COG0168 extracted from all MAGs. Within a substantial portion of MAGs, TrkH served as the transmembrane subunit of the Trk-type K^+^ transport system, rather than its homologous counterpart, TrkG. This observation implied that TrkH likely dominated as the transmembrane subunit in estuarine ecosystems, regardless of salinity category. Furthermore, the selection of transmembrane subunits of the Trk-type K^+^ transport system was likely to be closely related to the taxonomy. This was illustrated by the TrkG subunit being annotated in all MAGs of *Cyanobacteria*, and the TrkH subunit being almost universally annotated in MAGs of *Actinobacteriota* and *Proteobacteria* (Fig. 5e).

#### The “salt-out” strategy and the regulation of water channel activity

The “salt-out” strategy is an alternative and widely adopted approach employed by microorganisms to adapt to high-salinity habitats. This strategy entails the biosynthesis and accumulation of compatible solutes, thereby facilitating the maintenance of osmotic equilibrium and circumventing the detrimental impacts of excessive salt. COG0591, COG0477, and COG1115 were recognized as contributors to the “salt-out” strategy, facilitating the uptake of compatible solutes, such as glycine betaine, proline, and alanine, from the external environment into the cell [66, 67]. Regulating water transport across membranes is also a way for microorganisms to prevent osmotic damage. According to annotations in the eggNOG v5.0, KO, and GO databases, COG0580 is linked to GO: 0015250, and its function is to enable the facilitated diffusion of water [68–70].

##### The “salt-out” strategy and the regulation of water channel activity of the estuarine microbial community

In the context of the “salt-out” strategy, metagenomic analyses unveiled distinctive trends in relative abundance of the three relevant COGs. COG0591 (Fig. 6a, *R*^2^ = 0.6467, *p* = 0.003077) exhibited an increase in relative abundance with rising environmental salinity, while COG0477 (Fig. 6b, *R*^2^ = 0.6608, *p* = 0.002599) displayed a declining pattern. Furthermore, COG1115 (Fig. 6c, *R*^2^ = 0.8093, *p* = 0.0002428) demonstrated a significant increase in relative abundance. Regarding the regulation of water channel activity, COG0580 presented a decreasing trend in relative abundance with increasing salinity (Fig. 6d, *R*^2^ = 0.4783, *p* = 0.01603).

In the stenohaline MAGs, the relative abundance of COG0591 was significantly higher in the high-salinity category than in the low-salinity category, and a significant difference was observed between the intermediate- and low-salinity categories (Fig. 6e). COG0477 exhibited an opposite trend, aligning with the result obtained from the metagenomic perspective (Fig. 6b). Specifically, this COG demonstrated a significantly higher relative abundance in the low-salinity category compared with the intermediate- and high-salinity categories, with the intermediate category also showing higher abundance than the high-salinity category (Fig. 6f). The relative abundance of COG1115 was higher in the high- and intermediate-salinity categories compared to the low-salinity category, akin to COG0591 (Fig. 6g). Despite the Kruskal–Wallis rank-sum test indicating a significant difference in relative abundance across the three stenohaline categories for COG0580, pairwise comparisons revealed statistical significance only between MAGs categorized as intermediate- and low-salinity (Fig. 6h).

##### The “salt-out” strategy and the regulation of water channel activity of the estuarine dominant microbial phyla

In the stenohaline MAGs of the phyla *Actinobacteriota* and *Proteobacteria*, the relative abundances of the SOR COGs showed no significant difference across the three stenohaline categories (Additional file 1: Supplementary Figure S5a, S5b, and S5c). In the phylum *Actinobacteriota*, both COG0591 and COG1115 exhibited negligible relative abundances, as they were annotated in only one or two MAGs of the low- and intermediate-salinity categories (Additional file 1: Supplementary Figure S5a and S5c). On the other hand, all SOR COGs were extensively annotated in the phylum *Proteobacteria*, with COG0477 displaying a negative correlation with salinity and COG1115 showing a positive correlation (Additional file 1: Supplementary Figure S5a, S5b and S5c). COG0477 was annotated in the predominant microbial phyla of the PRE, with a high relative abundance being observed in the phylum *Actinobacteriota* (Fig. 3, Additional file 1: Supplementary Figure S5b). The relative abundance pattern of COG0580 was also analyzed within those two dominant microbial phyla. The results revealed that while there was no significant difference between *Proteobacteria* stenohaline MAGs, there was a significant difference between *Actinobacteriota* stenohaline MAGs of low- and high-salinity categories. In the phylum *Actinobacteriota*, the relative abundance of COG0580 was higher in the low-salinity category, whereas in the phylum *Proteobacteria*, it was higher in the high-salinity category (Additional file 1: Supplementary Figure S5d).

### Genes conferring resistance to environmental stresses beyond salinity tolerance

Microorganisms have developed various mechanisms that allow them to adapt to a wide range of environmental stresses including salinity. Here, several COGs related to microbial adaptation to abiotic factors other than salinity were identified (Fig. 3a, Additional file 2: Supplementary Table S9 and S10). These COGs were considered to be associated with heavy metal resistance (COG2217, COG0861, COG2059), drug resistance (COG2409), organic solvent resistance (COG0767), and sulfate metabolism (COG1613). Metagenomics showed that the relative abundances of these COGs decreased as salinity increased (Additional file 1: Supplementary Figure S6a-c, g-i). This trend was also reflected in the stenohaline MAGs, with the relative abundances of these COGs in the low-salinity category often higher than those in the intermediate- or high-salinity categories (Additional file 1: Supplementary Figure S6d–f, j–l). These results indicated that in areas closer to land with lower salinities, environmental stresses, such as high concentrations of heavy metals, drugs, and organic solvents, may cause changes in the functional characteristics of the microbial communities [71]. COG1613 (sulfate transport) was more abundant in the areas of lower salinities, which may be an adaptive strategy for microorganisms to cope with low oxygen condition in the low-salinity environment, with sulfate acting as an electron acceptor to sustain microbial life.

## Discussion

In this study, we applied metagenomics to explore the taxonomic and functional profiles of the microbial communities across the salinity gradient in the PRE, a typical subtropical estuary characterized by its short residence time. Furthermore, we aimed to unveil the mechanisms of estuarine microorganisms to cope with salinity stress, while also shedding light on the specific salinity adaptation strategies employed by the dominant taxa in the PRE.

The characteristics of the estuarine microbial communities are shaped by both salinity and hydrodynamic conditions. The residence time of the PRE was estimated to range from 3 to 12 days, which was shorter than that of other water bodies, such as the Baltic Sea (3 to 30 years) [13, 31]. However, it was comparable to the Columbia River Basin, where the water residence time was estimated to be an average of 1 to 2 days [12]. Salinity can exert a crucial influence on natural selection. This is evidenced by the differences in microbial community composition observed along the salinity gradient (Fig. 1a). Concerning the functional traits of the microbial communities, the low-salinity metagenomes formed a cluster separate from the intermediate- and high-salinity metagenomes, while the latter two clustered together. This pattern is consistent with that observed in the Columbia River short residence-time estuary and underscores the significant role of salinity in shaping the functional characteristics of the microbial communities, yet the influence of hydrodynamic conditions should not be underestimated in short residence-time estuaries [17]. In particular, active water exchange and short spatial distance may be contributing factors. Thus, we postulate that the microbial communities in intermediate- and high-salinity environments may share a great proportion of genes associated with the survival mechanisms required for living in the dynamic near-shore ecosystem and that insights into salinity adaptation mechanisms may be gleaned from studying specific functional genes. The findings of this study support a previous hypothesis, suggesting that estuaries with short residence times—typically spanning only a few days—undergo significant taxonomic shifts along the salinity gradient. Despite these shifts, changes at the functional level appear to be less pronounced [17].

The K^+^ uptake-based “salt-in” strategy may be the key mechanism allowing estuarine microorganisms to adapt to salinity stress in the PRE, especially for the two major estuarine phyla *Actinobacteriota* and *Proteobacteria*. In our study, over a quarter of the selected COGs were categorized as “inorganic ion transport and metabolism,” indicating the crucial role of this process for microbial survival and proliferation in the estuarine region (Additional file 2: Supplementary Table S8) [72]. In terms of osmoregulation, representative COGs were identified for both “salt-in” and “salt-out” strategies, with the most important COG being related to the “salt-in” strategy (COG0168). Interestingly, of the four SIR COGs, three were associated with K^+^ transport, including COG0168, which emerged as the top-ranking COG according to the Boruta algorithm (Fig. 3a, Additional file 2: Supplementary Table S8). Previous metagenomic studies have shown that K^+^ plays a role in the osmoregulation of the freshwater planktonic microbial communities [73, 74]. Here, we highlight that K^+^ uptake is also crucial for the adaptability of nearshore microorganisms to salinity stress, as demonstrated by *Actinobacteriota* and *Proteobacteria*, the two major microbial phyla inhabiting estuaries (Fig. 5). Moreover, the presence of at least one K^+^ transport system in nearly all euryhaline MAGs underscores the important role of K^+^ in microbial adaptation to osmotic stress fluctuation (Fig. 3b, Additional file 2: Supplementary Table S10) [75–77].

Fortunato et al. revealed that the abundance of Na^+^/H^+^ antiporters increased with rising salinity, suggesting a potential role in microbial adaptation to salinity in estuarine ecosystems [17]. However, no related genes were identified in our study. Although the concentration of Na^+^ is higher than that of K^+^ in the marine environment, excessive accumulation of Na^+^ has been found to be toxic to the cell. Thus, when salinity is elevated, microorganisms are unlikely to over accumulate Na^+^ to maintain osmolality [7, 75]. The preferential accumulation of K^+^ in cells can be attributed to the compatibility of this cation with water and protein structures. These advantages persist even at high concentrations due to its ion radius, the magnitude of its surface electric field, and its ability to form a unique hydration shell [75]. However, the possibility that Na^+^ can partially substitute for K^+^ within a range of concentrations cannot be ruled out, especially when K^+^ is lacking [73–75].

The Trk-type K^+^ transport system is suggested to be the primary transport system for K^+^ uptake by microorganisms in the PRE. In our study, two members of the K^+^ transporter superfamily (SKT)—specifically, the Trk-type K^+^ (COG0168) and Kup-type K^+^ (COG3158) transport systems—were selected for analysis and exhibited distinct and opposite abundance patterns (Figs. 3a and 4a, b, e, f) [75, 77–79]. A previous study has proposed that the Trk-type K^+^ transport system was critical for microbial adaptation to high-salinity stress in coastal sediments and soils [80]. This study suggests that the Trk-type K^+^ transport system may serve as the primary K^+^ transporter for microbial adaptation to salinity stress in surface water of short residence-time estuaries. This hypothesis is supported by shreds of evidence, including the top-ranked importance of the Trk-type K^+^ transport system, as determined by the Boruta algorithm, as well as observing similar patterns of increased relative abundance of this system with escalating salinity across the metagenomes, stenohaline MAGs, and dominant phyla (Figs. 4a, e, and 5a). It has been found that the transcriptional levels of the two membrane transporters (TrkH and TrkG) of the Trk-type K^+^ transport system were not significantly affected by the concentration of NaCl (0–1000 mM) [81]. Therefore, the increased relative abundance of the Trk-type K^+^ transport system with rising salinity may indicate a response of the estuarine microbial communities to osmotic stress. The widespread distribution of the Trk-type K^+^ transport system in the MAGs (both *Archaea* and *Bacteria*) supports the idea that it may be constitutive in the genomes of estuarine microorganisms that live in intermediate- to high-salinity habitats (Additional file 2: Supplementary Table S10). The importance of the transmembrane subunit TrkH of the Trk-type K^+^ transport system is also emphasized, suggesting its potential, rather than TrkG, as a key component of the Trk-type K^+^ transport system in allowing estuarine microorganisms to cope with salinity stress (Fig. 5e). This is possibly due to TrkH-mediated K^+^ uptake being unaffected by Na^+^ in the estuarine aquatic environment. A potential explanation for TrkG being annotated in all MAGs of *Cyanobacteria* is that the intake of Na^+^ is beneficial for their growth, as TrkG transports not only K^+^ but also Na^+^[81].

Typically, an increase in salinity is expected to cause an increase in the relative abundances of COGs associated with the “salt-in” or “salt-out” strategies that enhance microbial osmotic stress tolerance. However, our results demonstrated decreases in the relative abundances of COG3158 and COG0477 as salinity increased, both in the metagenomes and the stenohaline MAGs (Figs. 4b, and 6b, f). Additionally, we noted a significantly lower relative abundance of COG0038 in the high-salinity category compared with the intermediate-salinity category stenohaline MAGs (Fig. 4h). Although both the Trk-type and Kup-type K^+^ transport systems were considered low-affinity K^+^ transport systems and probably function by H^+^ symport, salinity may still exert selective pressure on them [76, 77]. It has been reported that the Kup-type K^+^ transport system (COG3158) was more effective at transporting K^+^ under low pH condition and that its activity was inhibited under elevated osmolarity [82, 83]. Thus, the negative correlation observed between the relative abundance of the Kup-type K^+^ transport system and salinity could be attributed to the lower pH of the low-salinity samples, as compared with that of the intermediate- and high-salinity samples. Another possible explanation involves high external osmotic stress. This stress could potentially suppress the Kup-type K^+^ transport system, thereby rendering Kup-dependent microorganisms unable to adapt to high-salinity environments. As a result, this could lead to a decrease in the relative abundance of this functional gene under such condition. COG0477 represents the ProP transporter, which can transport common osmoregulatory compounds, such as proline betaine, glycine betaine, and ectoine [48, 66]. In contrast to the overall stenohaline MAGs, COG0477 did not show any significant difference in relative abundance between the three stenohaline categories in the MAGs of the two dominant phyla, *Actinobacteriota* and *Proteobacteria* (Additional file 1: Supplementary Figure S5b). Moreover, COG0477 generally showed a substantially high relative abundance in the MAGs affiliated with *Actinobacteriota* (Fig. 3b). We postulate that the inverse correlation between the relative abundance of COG0477 and salinity is closely related to the succession of the estuarine dominant microbial community. Specifically, under high-salinity conditions, the relative abundances of MAGs affiliated with *Actinobacteriota* decreased, and those MAGs harbored a high abundance of COG0477 (Figs. 1a and 2a). COG0038, annotated as Cl^−^ channel, exhibited a significantly higher relative abundance in MAGs of the intermediate-salinity category compared with the two other salinity categories, implying its potential role in regulating osmotic balance that may be limited to a specific salinity range (Fig. 4h).

In response to osmotic imbalance, water immediately rushes into the cell, from low solute concentrations to high solute concentrations, until the salt concentration equilibrates across the membrane [64]. The metagenomic analysis showed a decreasing trend in the relative abundance of COG0580 with increasing salinity, indicating that high-salinity-adapted microorganisms may decrease the relative abundance of the water channel activity regulation gene to maintain intracellular osmotic pressure (Fig. 6d). However, a similar abundance pattern was not observed in the stenohaline MAGs, even exhibiting opposite trends in *Actinobacteriota* and *Proteobacteria* stenohaline MAGs (Fig. 6h, Additional file 1: Supplementary Figure S5d). This discrepancy is likely due to the fact that controlling water channel activity for osmoregulation may not be universally employed by all microorganisms, or to the possibility that the water permeability of the cytoplasmic membrane may be sufficiently high [84].

It is noteworthy that the results obtained through metagenomics illustrate the genomic characteristics of microorganisms and their potential abilities to respond to salinity stress. The methodology and objectives of this study were not specifically designed to cover all genes related to microbial salinity adaptation, especially those genes that may not show significant differences in relative abundance across different salinity categories yet remain crucial to microbial salinity adaptation. In the future, as more microbial pure cultures are obtained and other omics approaches, such as metatranscriptomics and metaproteomics are integrated, along with the utilization of more advanced algorithms and research techniques, a more comprehensive understanding of microbial adaptation to salinity will be achieved.

## Conclusions

The present study aimed to explore the impact of salinity on the microbial communities in the nearshore water column and the salinity adaptation characteristics of microorganisms from a community perspective. The phyla *Actinobacteriota* and *Proteobacteria* constituted the main microbial groups in the PRE, and spatial heterogeneity in microbial community composition was observed along the river-to-ocean continuum. Salinity is supposed to influence the functional traits of the microbial communities in the estuarine ecosystem. However, in the PRE, the impact of salinity may be mitigated by active water exchange. Within the short residence-time estuarine ecosystem, the transport and metabolism of inorganic ions were found to be crucial for the microorganisms to thrive. These estuarine microorganisms were endowed with the ability to adapt to salinity stress through multiple strategies, including K^+^ uptake, transport of compatible solutes, and regulation of water efflux/influx. Herein, we highlighted the pivotal role of the K^+^ uptake-based “salt-in” strategy in microbial adaptation to salinity stress, most notably within the dominant *Actinobacteriota* and *Proteobacteria* phyla of the estuarine microbial communities. Furthermore, the importance of the Trk-type K^+^ transport system in facilitating this adaptive process was emphasized. Taken together, this study provides valuable insights into microbial community-level and population-level adaptation strategies to salinity stress. Building upon previous contributions, our findings deepen the current understanding about the spatial distribution of microorganisms and their survival mechanisms within the estuarine ecosystem by linking specific genes to microbial salinity adaptation. Moreover, continued advancements in multi-omics approaches, more comprehensive gene annotation, and global research efforts are expected to provide further insights into the specific pathways and evolutionary patterns of salinity adaptation by microorganisms with different lifestyles, niches, and taxonomic affiliations.

### Supplementary Information

**Additional file 1:** **Supplementary Figure S1.** Sampling stations from the Pearl River to the northern South China Sea. The insert shows an enlarged view of part of the studied areas. Sampling stations are represented as dots colored according to their salinity categories. **Supplementary Figure S2.** **(a)** Relative abundances of classified 16S miTags in the metagenomes. Source data are provided in Additional file 2: Supplementary Table S7. **(b)** NMDS conducted on the Bray-Curtis dissimilarities based on the taxonomic profiles of the contigs (annotated by Kaiju software). The ellipses in the plot mark the 90% confidence interval for MAGs grouped by the salinity category. The points representing PR5 and PR7 overlap in the figure. **(c)** Complete linkage hierarchical clustering based on the taxonomic profiles of the contigs (annotated by Kaiju software) using Bray-Curtis dissimilarities. The salinity categories of the metagenomes are denoted using markers of different colors and shapes. Different colored boxes indicate the grouping of these branches into four main subdivisions. **Supplementary Figure S3.** **(a)** NMDS conducted on the Bray-Curtis dissimilarities based on the relative abundances of stenohaline MAGs across different salinity categories. The ellipses in the plot mark the 90% confidence interval for MAGs grouped by the stenohaline salinity category. The ANOSIM test was used to assess the differences in relative abundances of stenohaline MAGs between **(b)** high-, intermediate-, and low-salinity categories; **(c)** high- and low-salinity categories; **(d)** intermediate- and low-salinity categories; and **(e)** high- and intermediate-salinity categories. Dis. rank: rank of dissimilarity entry. **Supplementary Figure S4.** Dendrogram for complete-linkage hierarchical clustering of the relative abundances of all COGs in all 127 MAGs using the Bray-Curtis dissimilarity. Each branch in the dendrogram represents a MAG and each color represents a phylum. Dots of different colors stand for different salinity categories. **Supplementary Figure S5.** Relative abundances of **(a–c)** the SOR COGs and **(d)** COG0580 in stenohaline MAGs affiliated with *Actinobacteriota* and *Proteobacteria*. All *p* values are within the 95% confidence interval of bootstrap tests (solid blue dots). Number of MAGs involved in statistical calculations: low-salinity category *Actinobacteriota*, *n*=17; low-salinity category *Proteobacteria*, *n*=11; intermediate-salinity category *Actinobacteriota*, *n*=10; intermediate-salinity category *Proteobacteria*, *n*=16; high-salinity category *Actinobacteriota*, *n*=3; high-salinity category *Proteobacteria*, *n*=13. Boxplot components: center lines, medians; box limits, 25^th^ and 75^th^ percentiles; whiskers, 1.5× interquartile range from the 25^th^ and 75^th^ percentiles; red points, outliers. Statistical significance symbols: ****p≤0.0001; ***p≤0.001, **p≤0.01, *p≤0.05, ns *p*>0.05 (Kruskal–Wallis rank-sum test with Dunn’s multiple comparison test). Source data are provided in Additional file 2: Supplementary Table S10. **Supplementary Figure S6.** Relative abundances of COGs involved in microbial adaptation to other stresses at the metagenomic **(a–c, g–i)** and MAG **(d–f, j–l)** levels. All R-squared values and *p*-values are within the 95% confidence interval of bootstrap tests (solid blue dots). Number of MAGs involved in statistical calculations: low-salinity category, *n*=33; intermediate-salinity category, *n*=36; high-salinity category, *n*=44. Boxplot components: center lines, medians; box limits, 25^th^ and 75^th^ percentiles; whiskers, 1.5× interquartile range from the 25^th^ and 75^th^ percentiles; red dots, outliers. Statistical significance symbols: ****p≤0.0001; ***p≤0.001, **p≤0.01, *p≤0.05, ns *p*>0.05 (Kruskal–Wallis rank-sum test with Dunn’s multiple comparison test). Source data are provided in Additional file 2: Supplementary Table S10.

**Additional file 2:** **Supplementary Table S1.** Physicochemical parameters and metagenomic data sizes. **Supplementary Table S2.** Shotgun metagenomics sequencing statistics and COG annotation for metagenomes. **Supplementary Table S3.** Characteristics of the 127 MAGs reconstructed in this study. **Supplementary Table S4.** Raw relative abundances of 127 MAGs reconstructed in this study and their taxonomy and salinity categories. **Supplementary Table S5.** Proportion of sequences recruited by the MAGs from each metagenomic sample and the co-assembly. **Supplementary Table S6.** Taxonomic annotation results based on metagenomic contigs and Shannon-Wiener diversity indices. **Supplementary Table S7.** Statistics of ribosomal gene sequences identified and annotated from the metagenomes. **Supplementary Table S8.** COG functions that significantly segregated across MAGs of different salinity categories selected by the Boruta random forest algorithm based on 8000 permutations. **Supplementary Table S9.** Relative abundances of COGs (%) related to microbial adaptation to the estuarine environment in the metagenomes. **Supplementary Table S10.** Relative abundances of COGs (%) related to microbial adaptation to the estuarine environment in the MAGs.

## Data Availability

All sequence data (metagenomic dataset and MAGs) presented in this article is publicly available in the repository of NCBI under Bioproject number PRJNA878949.

## Abbreviations

ANOSIM: Analysis of similarities
COG: Clusters of Orthologous Group
MAG: Metagenome-assembled genome
NMDS: Non-metric multidimensional scaling
PRE: Pearl river estuary
RDP: Ribosomal database project
SIR COG: “Salt-in” strategy–related COG
SOR COG: “Salt-out” strategy–related COG
