# Supplementary figures and images for: Metagenomic insights into microbial adaptation to the salinity gradient of a typical short residence-time estuary
Source: Microbiome. 2024 Jun 25;12:115. doi: 10.1186/s40168-024-01817-w (PMC11200988; doi:10.1186/s40168-024-01817-w)

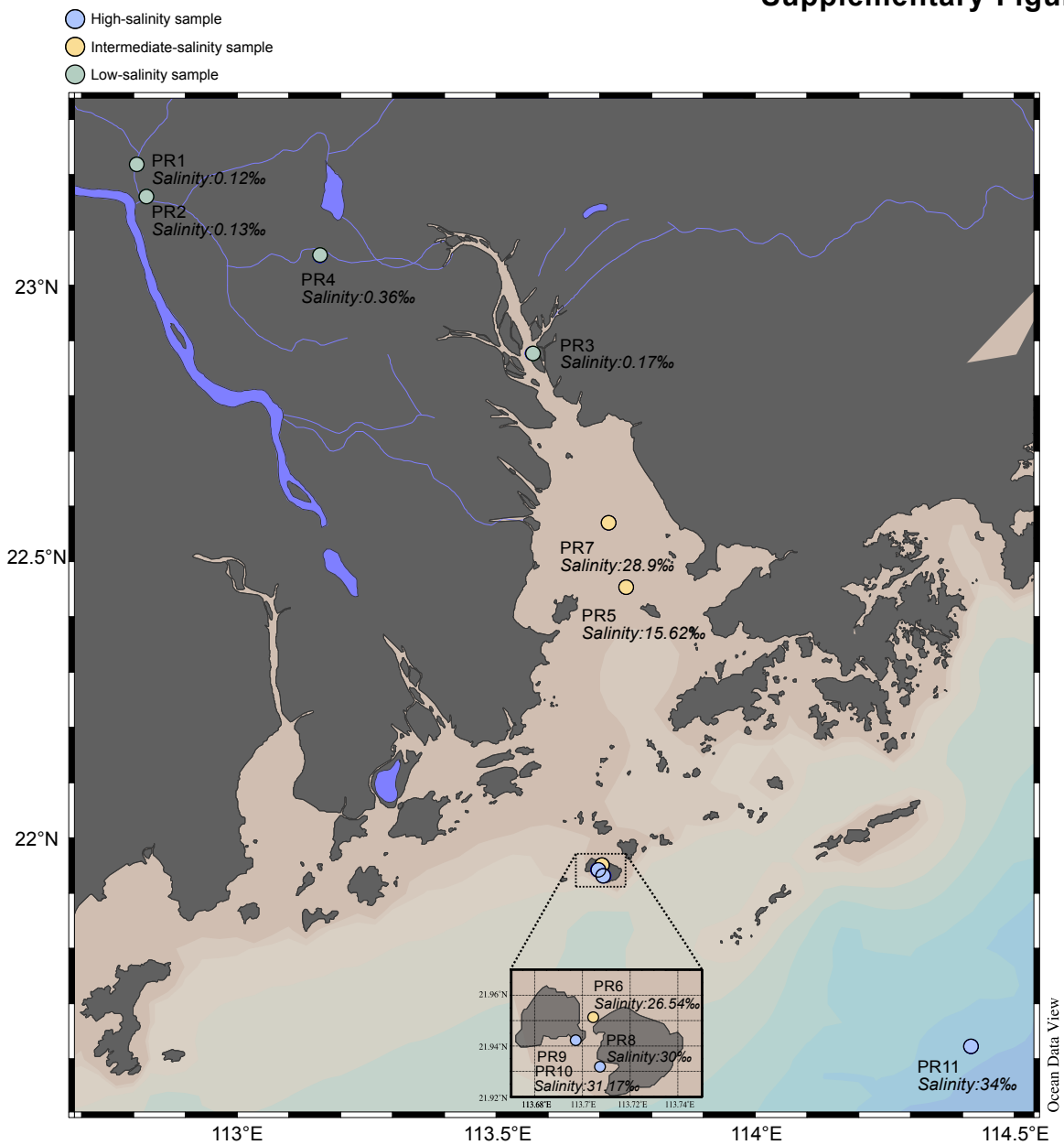

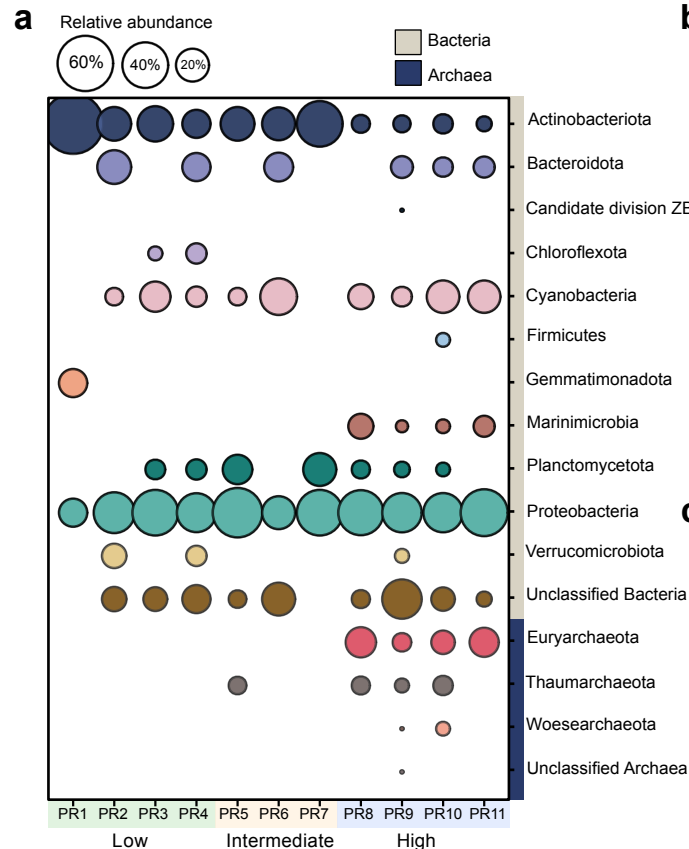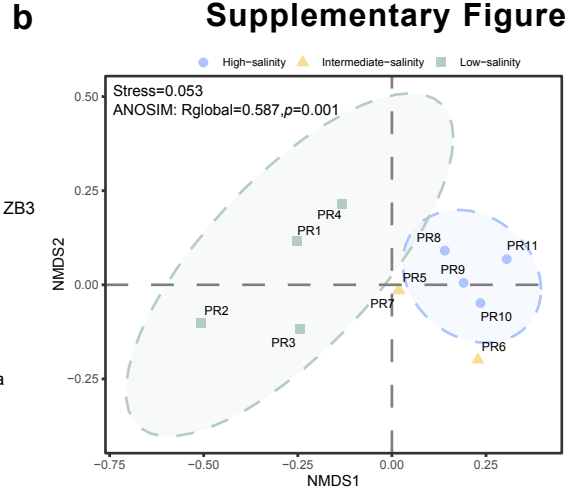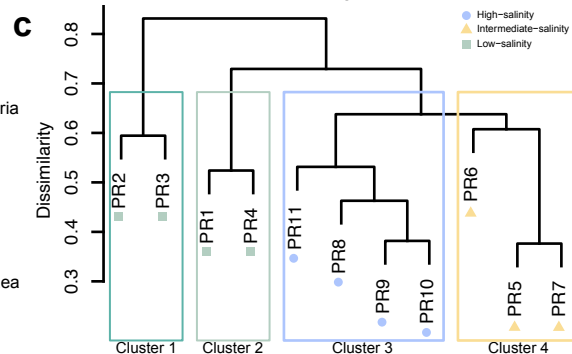

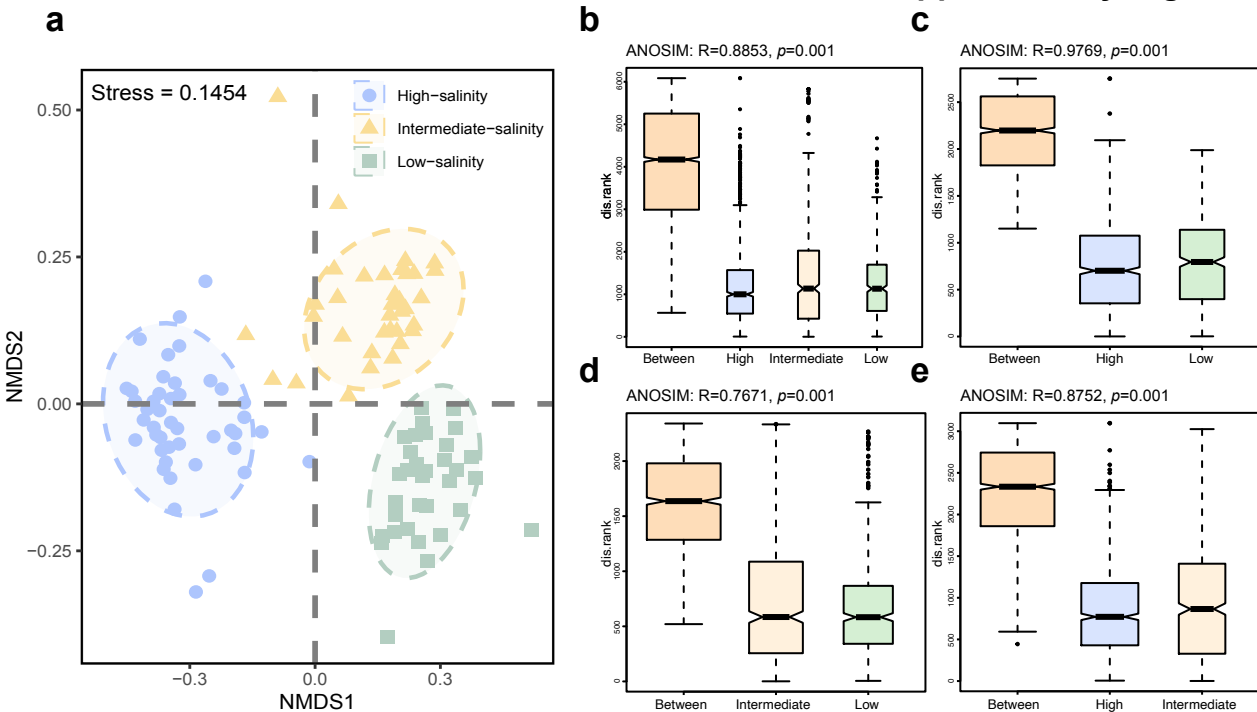

# Supplementary Figure S4

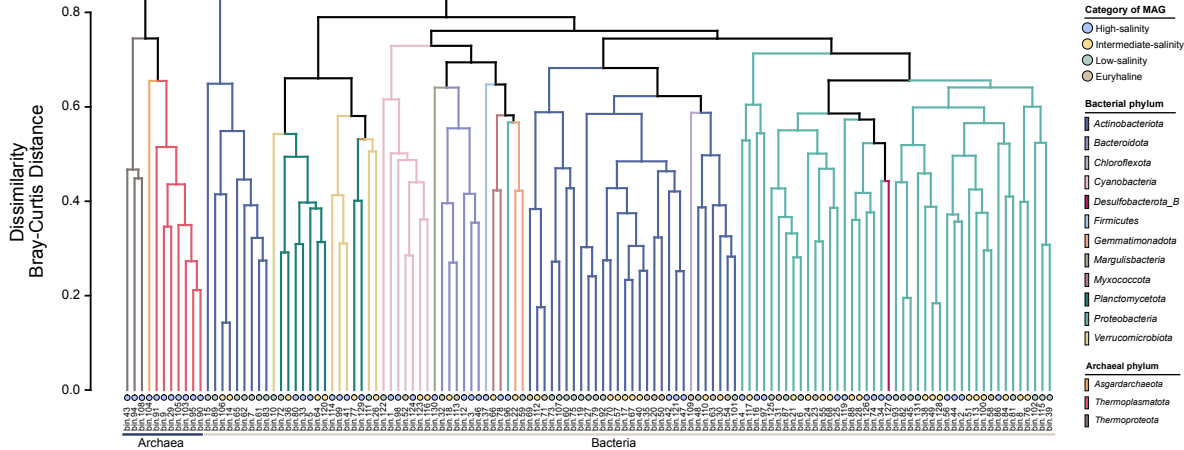

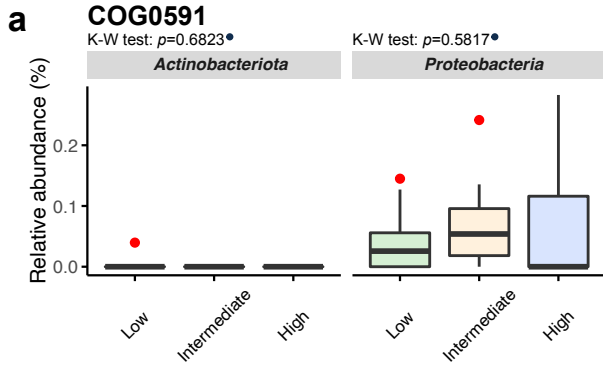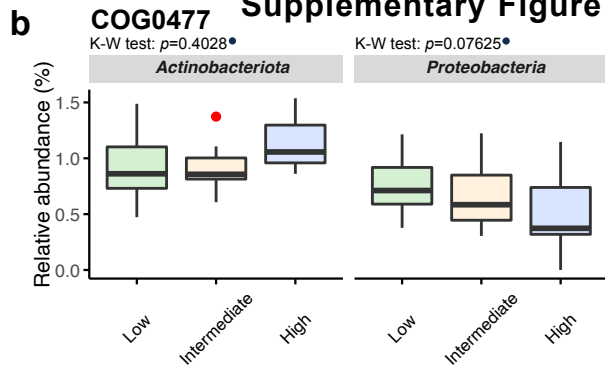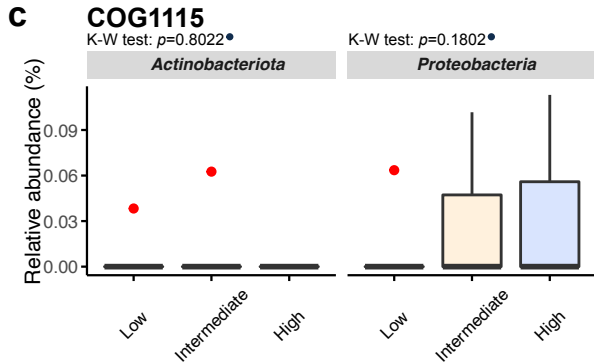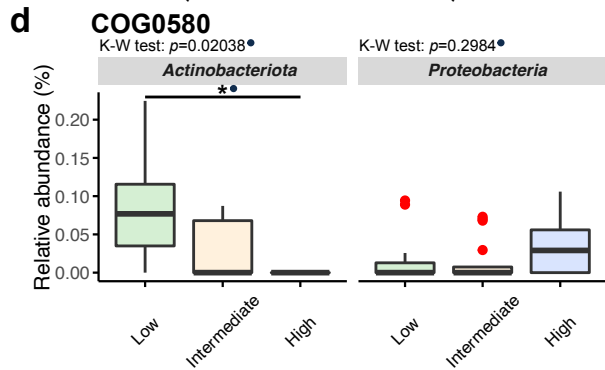

● Statistical value falls within 95% bootstrap confidence interval

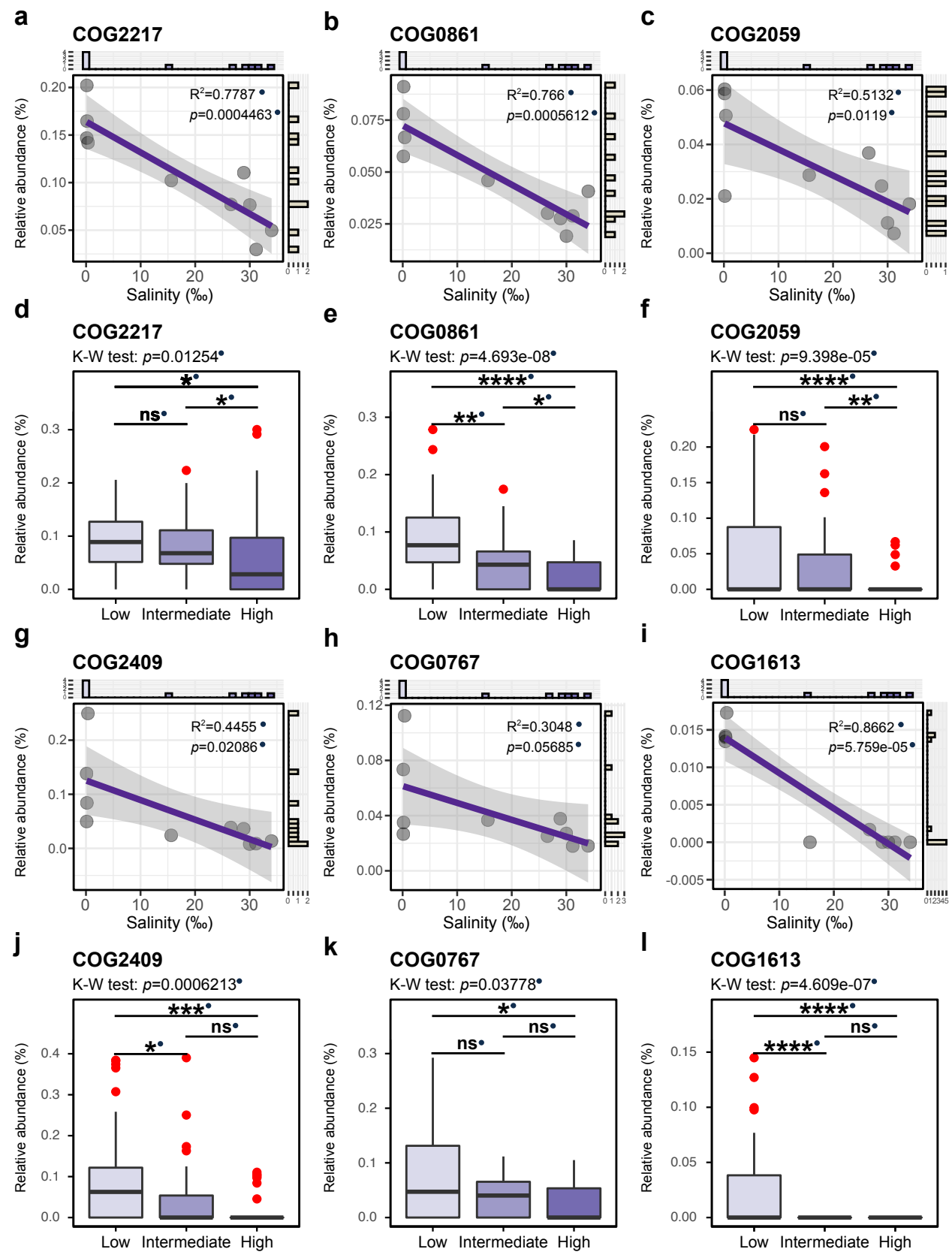

Supplement: Supplementary file 2 — Additional file 1: Supplementary Figure S1. Sampling stations from the Pearl River to the northern South China Sea. The insert shows an enlarged view of part of the studied areas. Sampling stations are represented as dots colored according to their salinity categories. Supplementary Figure S2. (a) Relative abundances of classified 16S miTags in the metagenomes. Source data are provided in Additional file 2: Supplementary Table S7. (b) NMDS conducted on the Bray-Curtis dissimilarities based on the taxonomic profiles of the contigs (annotated by Kaiju software). The ellipses in the plot mark the 90% confidence interval for MAGs grouped by the salinity category. The points representing PR5 and PR7 overlap in the figure. (c) Complete linkage hierarchical clustering based on the taxonomic profiles of the contigs (annotated by Kaiju software) using Bray-Curtis dissimilarities. The salinity categories of the metagenomes are denoted using markers of different colors and shapes. Different colored boxes indicate the grouping of these branches into four main subdivisions. Supplementary Figure S3. (a) NMDS conducted on the Bray-Curtis dissimilarities based on the relative abundances of stenohaline MAGs across different salinity categories. The ellipses in the plot mark the 90% confidence interval for MAGs grouped by the stenohaline salinity category. The ANOSIM test was used to assess the differences in relative abundances of stenohaline MAGs between (b) high-, intermediate-, and low-salinity categories; (c) high- and low-salinity categories; (d) intermediate- and low-salinity categories; and (e) high- and intermediate-salinity categories. Dis. rank: rank of dissimilarity entry. Supplementary Figure S4. Dendrogram for complete-linkage hierarchical clustering of the relative abundances of all COGs in all 127 MAGs using the Bray-Curtis dissimilarity. Each branch in the dendrogram represents a MAG and each color represents a phylum. Dots of different colors stand for different sal [file 40168_2024_1817_MOESM1_ESM.pdf]
